# Higher Algebraic K-Theory of Causality

**DOI:** 10.3390/e27050531

**Published:** 2025-05-16

**Authors:** Sridhar Mahadevan

**Affiliations:** Adobe Research, 345 Park Avenue, San Jose, CA 95110, USA; smahadev@adobe.com

**Keywords:** causal inference, structural causal models, Bayesian networks, symmetric monoidal categories, PROPs, higher algebraic K-theory, homotopy, classifying spaces

## Abstract

Causal discovery involves searching intractably large spaces. Decomposing the search space into classes of observationally equivalent causal models is a well-studied avenue to making discovery tractable. This paper studies the topological structure underlying causal equivalence to develop a categorical formulation of Chickering’s transformational characterization of Bayesian networks. A homotopic generalization of the Meek–Chickering theorem on the connectivity structure within causal equivalence classes and a topological representation of Greedy Equivalence Search (GES) that moves from one equivalence class of models to the next are described. Specifically, this work defines causal models as propable symmetric monoidal categories (cPROPs), which define a functor category $C^{P}$ from a coalgebraic PROP *P* to a symmetric monoidal category C. Such functor categories were first studied by Fox, who showed that they define the right adjoint of the inclusion of Cartesian categories in the larger category of all symmetric monoidal categories. cPROPs are an algebraic theory in the sense of Lawvere. cPROPs are related to previous categorical causal models, such as Markov categories and affine CDU categories, which can be viewed as defined by cPROP maps specifying the semantics of comonoidal structures corresponding to the “copy-delete” mechanisms. This work characterizes Pearl’s structural causal models (SCMs) in terms of Cartesian cPROPs, where the morphisms that define the endogenous variables are purely deterministic. A higher algebraic K-theory of causality is developed by studying the classifying spaces of observationally equivalent causal cPROP models by constructing their simplicial realization through the nerve functor. It is shown that Meek–Chickering causal DAG equivalence generalizes to induce a homotopic equivalence across observationally equivalent cPROP functors. A homotopic generalization of the Meek–Chickering theorem is presented, where covered edge reversals connecting equivalent DAGs induce natural transformations between homotopically equivalent cPROP functors and correspond to an equivalence structure on the corresponding string diagrams. The Grothendieck group completion of cPROP causal models is defined using the Grayson–Quillen construction and relate the classifying space of cPROP causal equivalence classes to classifying spaces of an induced groupoid. A real-world domain modeling genetic mutations in cancer is used to illustrate the framework in this paper.

## 1. Introduction

**Figure 2:**
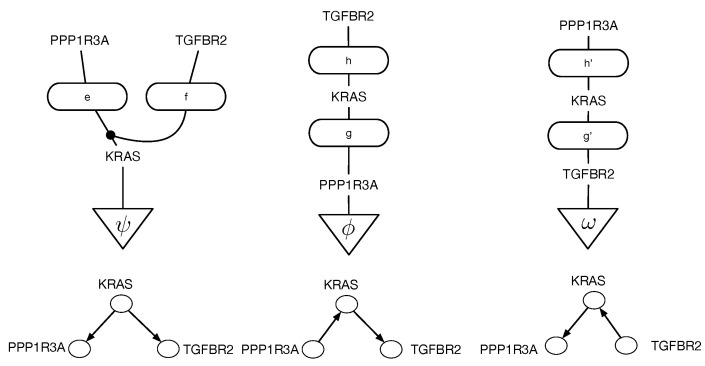
Equivalence classes of causal DAGs and cPROP string diagrams on 3 variables from a pancreatic cancer domain described in Section 2 in more detail. KRAS, TGFBR2, and PPP1R3A define three genes which are mutated in many pancreatic cancer tumors, and the challenge in causal modeling is to discover a partial ordering of the gene mutations. For each DAG at the bottom, the corresponding cPROP string diagram is shown above. The three DAGs shown form a single equivalence class, which implies the three string diagrams also are equivalent. The causal discovery method GES [3], described in Section 3, searches in the space of such equivalence classes.

Causal discovery using methods such as FCI [1] or IC [2], as well as the many variants and extensions of these classic methods developed over the past several decades [3,4,5,6,7], involves searching super-exponential spaces as the number of causal DAGs grows extremely large in the number of variables. To reduce this intractable search space, it is often possible to form equivalence classes of observationally equivalent causal models (see Figure 2). There are approximately $10^{18}$ DAG models on just 11 labeled variables. To make matters worse, DAG models capture only a tiny portion of the space because for n=4, there are 18,300 conditional independence structures, but DAG models capture only roughly 1% of this space! As we illustrate later in Section 2, constructing causal models for pancreatic cancer requires dealing with many thousands of potentially mutated genes that combine in a dozen known pathways, leading to a search space of causal models that can be astronomically large. More powerful models like integer-valued multisets (imsets) [8] that model conditional independences by mapping the powerset of all variables into integers grow even larger still (of the order of $2^{2^{n}}$). Representing this space efficiently with categorical representations like affine CDU categories [9] or Markov categories [10] will require defining equivalence classes over string diagrams to combat this curse of dimensionality. This challenge motivates the need for a deeper categorical understanding of the equivalence classes of observationally indistinguishable models [11]. While allowing for arbitrary interventions on causal models enables accurate identification [6,7], such interventions are rarely practical in the real world. Insights such as the Meek–Chickering theorem [3,12,13] allow a deeper understanding of connected paths among equivalent causal DAG models, which we propose to study using a homotopy framework in this paper.

**Figure 1:**
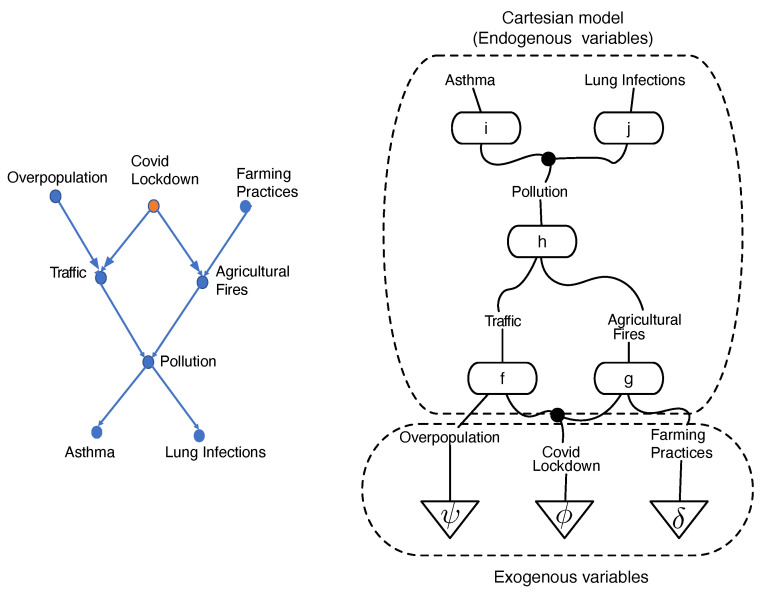
A Cartesian cPROP causal model that represents a structural causal model of pollution in New Delhi, India (based on original model defined in [14]). Here, following the usual custom in SCMs [2], the functions defining endogenous variables (f,g,h,i, and *j*) are deterministic, whereas the exogenous variables are defined by probability distributions (e.g., ψ:I→Overpopulation defines a distribution in a Markov category). In keeping with the usual practice [9,17], information flows from bottom to top in string diagrams, which is the opposite of DAG models. Also, nodes in DAG models represent variables, and the functions are implicitly defined by the edges. In string diagrams, the edges define variables, and the functions are explicitly represented as boxes. To fully specify a cPROP causal model, we have to define a functor that maps such a string diagram into a decomposable stochastic process, which is illustrated later in Figure 12.

To generalize the Meek–Chickering theorem to the categorical setting, some challenges need to be addressed. Figure 1 shows a string diagram representation of a pollution causal model first used in our previous paper on universal causality [14]. Such string diagrams are used in affine CD [9] and Markov categories [10]. As the number of causal models grows exponentially, so does the number of string diagrams, and to develop deeper insight into the underlying topological structure of causal equivalences, we introduce a coalgebraic theory of causal inference based on a categorical structure we call cPROP, which is defined as a functor category from a PROP [15] to a symmetric monoidal category [16].

To help motivate the need for cPROPs, note that in a causal model, variables are “reused” across different local causal mechanisms. A simple example is the DAG A←B→C, whose joint distribution P(A,B,C)=P(A|B)P(C|B) decomposes in a way that reflects the conditional independence structure of the DAG. Here, the variable *B* is used twice, and to make it accessible across multiple expressions, any such variable must be “copied”. Such a copy mechanism has been used in previous work on categorical causal models based on string diagrams [9,10,17], which have been referred to as “copy-delete-uniform” (CDU) categories. Here, “deletion” refers to the requirement that any distribution *P* can be marginalized to 1 by summing over all its values, which in categorical terms are modeled by a “delete” mechanism f:X→I (where *X* is some object that represents a distribution). cPROPs provide a way to define such an “internal” category over an external category that specifies such “copy” and “delete” mechanisms by modeling them as “comonoid” objects within a category.

Causal discovery poses some unique challenges for categorical modeling. Figure 2 illustrates the structure of causal equivalence classes on causal DAGs for a simple causal model in the pancreatic cancer domain described in Section 2. As first observed in Verma and Pearl [11], two DAGs are equivalent if their underlying skeletons (undirected graph structure ignoring edge directions) and V-structures X→Z←Y are the same. Our goal here is to build on the ideas in [3] on connected paths between observationally equivalent models, in particular the Meek–Chickering theorem, which we want to generalize to the categorical setting. As Chickering [3] notes, this theorem, which was originally a conjecture by Meek, implies that there exists a sparse search space, where each candidate model is connected to a small fraction of the total space, given a generative distribution that has a perfect map in a DAG defined over the observables. This property leads to the development of a greedy search algorithm that in the limit of training data can identify the correct model.

**Figure 3:**
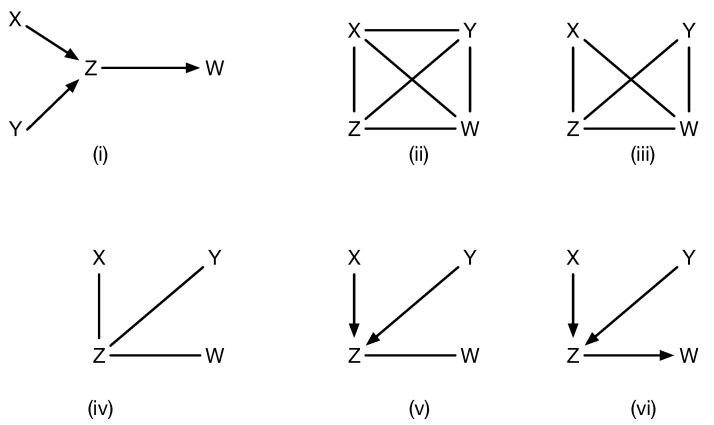
Example of causal discovery with the PC algorithm [5]. Panel (i) shows the original causal model, and panels (ii)–(vi) show the sequence of models constructed by PC.

In practice, existing causal discovery algorithms, such as PC [1] or IC [2] or their many extensions and variants, combine both directional and non-directional encoding of causal models. Specifically, a common assumption, such as in PC, is that given an unknown true causal model (shown in Figure 3 by panel (i)), the initial causal model (shown as (ii) in Figure 3) is an undirected graph connecting all variables to each other, which satisfies no conditional independences, and is progressively refined (panels (ii)–(vi) in Figure 3) based on conditional independence data and using edge orientation and propagation rules, such as the Meek rules [12]. For example, the initial stage is to simply check all marginal independences, and given that X⊥⊥Y, that eliminates the undirected edge between *X* and *Y*. However, each undirected edge between two vertices, say *A* and *B*, that needs to be eliminated due to conditional independence must be checked for increasingly large subsets (A⊥⊥B|C), and while methods like FCI and later enhancements [6,7] incorporate rather sophisticated methods to prune the space, this process remains computationally expensive, and its practicality remains in question, as in the real world, interventions on arbitrary separating sets [6] may be infeasible. While remarkable progress has been made over the past few decades (see [7] for a state of the art method), it still can be prohibitive and does not always end up with the right model. Edges that remain undirected are interpreted to indicate latent confounders.

This paper builds on the work of Fox [18], who studied functor categories mapping PROPs to symmetric monoidal categories in his PhD dissertation in 1976. Crucially, Fox [18] studied a particular functor category from a coalgebraic PROP to symmetric monoidal categories that defined a right adjoint from the category **MON** of all symmetric monoidal categories to **CART**, which is the category of all Cartesian categories. We use the term coalgebraic in the universal algebraic sense as used by Fox [18]. It differs from the modern interpretation as in [19]. In this sense, cPROPs are formally an algebraic theory in the sense of Lawvere [20].

Objects in a cPROP are functors mapping a PROP *P*—a symmetric monoidal category over natural numbers—to a symmetric monoidal category C. The structure PROP (for Products and Permutations) was originally introduced by Maclane [15], and it has seen widespread use in many areas such as modeling connectivity in networks [21,22]. A trivial example of a PROP is the free monoidal category Γ, whose objects can be interpreted as the natural numbers, the unit object is 0, and the tensor product is addition. More generally, a PROP *P* is a small monoidal category with a strict monoidal functor Γ→P that is a bijection on objects. A cPROP is a functor category $C^{P}$, where C is a symmetric monoidal category, where in addition there are usually some constraints placed on the specific PROP *P*.

As a simple example, we consider cPROPs where the PROP *P* is generated by a *coalgebraic* structure defined by the maps δ:1→2 and ϵ:1→0 satisfying a set of commutative diagrams. Such cPROPs are closely related to symmetric monoidal category structures used in previous work on categorical models of causality, probability, and statistics [10,14,23,24,25]. In particular, Markov categories [10,17] and affine CDU (“copy-delete-uniform”) categories used to model causal inference include a comonoidal “copy delete” structure corresponding to such a cPROP, which we note is distinctive in that “delete” has a uniform structure but “copy” does not, leading to a semi-Cartesian category.

In my previous work on universal causality [14], I proposed the use of simplicial sets, which provide a way to encode both directional and non-directional edges, as well as form the basis for topological realization for cPROPs and play a central role in higher-order *∞*-categories [26,27]. We study the classifying spaces [28] of cPROPs in this paper, showing that they provide deeper insight into the connections between different cPROP categories that correspond to Markov categories, such as **FinStoch** [17].

In particular, this work builds on longstanding ideas in abstract homotopy theory on modeling the equivalence classes of objects in a category [29] by mapping a category into a topological space, where (weak) equivalences can be modeled in terms of topological structures, such as homotopies. To make this more concrete, Jacobs et al. [9] modeled a Bayesian network as a CDU functor F:C→FinStoch between two affine CDU or Markov categories, with one specifying the graph structure of the model and the other modeling its semantics as an object in the category of finite stochastic processes defined as **FinStoch**. A CDU functor is a special type of cPROP functor. Two Bayesian networks modeled as cPROP functors that are observationally equivalent—such as A→B→C and A→B←C, since the edge B←C is a covered edge that can be reversed—induce a natural transformation $\tau :F_{1}\Rightarrow F_{2}$. Using the associated classifying spaces BC and BFinStoch, the natural transformation induces a homotopy between $F_{1}$ and $F_{2}$.

The idea of associating a topological space with a category goes back to Grothendieck but was popularized by Segal [28]: map a category C to a sequence of sets (or objects) $X_{0},X_{1},\ldots$, where the *k*-simplex $X_{k}$ represents composable morphisms of length *k*. A standard topological realization proposed by Milnor [30] constructs a topological CW complex out of simplicial sets. Segal called such a construction the classifying space BC of category C. This paper can be seen as an initial step in building a higher algebraic K-theory [31] for causal inference, using as a concrete example the study of classifying spaces of cPROPs. A 0-simplex in a simplicial cPROP would be defined by its objects X,Y,X⊗Y,…, which map to 0-cells in its classifying space. An example 2-simplex in a cPROP, such as I→X⊗Y→X, maps to a 2 cell or simplicial triangle.

This paper builds on the insight underlying Fox’s dissertation on universal coalgebras [18], which shows that the subcategory of coalgebraic objects in a monoidal category forms its Cartesian closure. The adjoint functor theorems show that cofree algebras—right adjoints to forgetful functors—exist in such cases. In particular, Fox’s theorem implies that cPROPs that come with a type of “uniform copy-delete” structure [32] are Cartesian symmetric monoidal categories, where the tensor product X⊗Y becomes a Cartesian product operation through natural transformations rather than the standard universal property. It is noted that Markov categories are semi-Cartesian because the comonoidal ${\mathrm{copy}}_{X}$ structure is not uniform, but only ${\mathrm{del}}_{X}$ is. However, they contain a subcategory of deterministic morphisms that induce a Cartesian category using the uniform copy delete structure. It is worth noting here that Pearl [2] has long advocated causality as being being intrinsically deterministic in his structural causal models (SCMs), where the role of probabilities is reflected in the uncertainty associated with exogenous variables that cannot be causally manipulated.

Here is a roadmap to the rest of the paper. To concretize the abstractions presented in the paper, it begins in Section 2 with an application to constructing causal models of pancreatic cancer [33,34,35]. Section 3 describes a concrete procedure for causal discovery called Greedy Equivalent Search (GES) [3,12] that uses a specific notion of causal equivalence based on a transformational characterization of Bayesian networks, which are generalized to a homotopical setting. GES is also illustrative of a broad class of similar algorithms. Numerous refinements are possible, including the ability to intervene on arbitrary subsets [6,7], which are overlooked in the interests of simplicity. Section 4 begins with an introduction to algebraic theories of the type proposed by Lawvere [20], a brief review of symmetric monoidal categories, and an introduction to PROPs and cPROPs. Functor categories mapping a PROP to a symmetric monoidal category are defined. The central result of Fox is reviewed, showing that the inclusion of all Cartesian categories CART in the larger category of all symmetric monoidal categories MON has a right adjoint, which is defined by a coalgebraic PROP functor category. This coalgebraic structure relates to the “uniform copy-delete” structure studied by [32]. In Section 5, the relationships between cPROPs with uniform copy and delete natural transformations and previous work on affine CDU categories [25] and Markov categories [17] are explored. Section 6 explores the relationship between Pearl’s structural causal models (SCMs) and Cartesian cPROPs defined by deterministic morphisms, exploiting the property that SCMs are defined by purely deterministic mappings from exogenous variables to endogenous variables. In Section 7, simplicial objects in cPROP categories are defined. Section 8 defines the abstract homotopy of cPROPs at a high level. Section 9 drills down into showing the homotopic structure of cPROP functors that represent Bayesian networks, which closely relates to the work on CDU functors [9]. Natural transformations in the functor category of Bayesian networks modeled as cPROPs using Yoneda’s coend calculus [16] are characterized, and an equivalence relationship among functors is defined. In particular, categorical generalizations of the definitions of equivalent causal models in [3,12] are presented, and a homotopic generalization of the well-known Meek–Chickering theorem for cPROPs is stated. Each edge reversal of a covered edge corresponds to natural transformation between its corresponding cPROP functor. This work formally characterizes the classifying spaces of cPROPs in terms of associative and commutative *H*-spaces [29]. Finally, the results of the previous section in Section 11 are combined, stating the main result that the Grayson–Quillen procedure applied to cPROP yields a category $C^{-1}C$ that represents a Grothendieck group completion of cPROP category C and whose connected components that define the 0th order homology (loop) space are isomorphic to the Meek–Chickering equivalence classes. In Section 12, a more advanced application of the framework to open games [36] and network economics [37] is defined, wherein both of these fields can be defined using symmetric monoidal categories and are therefore amenable to the approach given here. In Section 13, the paper is summarized, and an outline of a few directions for further work is given.

## 2. Causal Models of Genetic Mutations in Cancer

This section illustrates a real-world application that will serve as a running example to illustrate the causal framework: modeling mutations of genes for understanding the processes that underlie various types of cancer [33,38]. Cancer is an evolutionary disease that is generally characterized by an accumulating series of genetic mutations. The temporal order of these mutations can be viewed as a partially ordered set, or DAG model, which can be further modeled using specific temporal information with respect to when particular mutations occur. A wide range of causal models can be applied to this problem, such as *conjunctive Bayesian Networks* [34], which are a simpler class of Bayesian networks that exploit the property that mutations induce a partial ordering over genes, as they are irreversible.

Table 1 illustrates the general type of data that is available for many types of cancer, including colorectal cancer, pancreatic cancer, primary gliolastoma, etc. The 0,1 values in the table indicate whether a particular gene was mutated in a specific instance of a tumor. There is generally a partial ordering that defines the allowable sequences of mutations that are observed in many types of cancer. It is not the case that mutations occur in any order, and in most cancers, it is usually the case that the mutations form particular types of sequences. Modeling these posets through a causal model has been extensively studied in the literature [34,38]. We discuss one specific case study of pancreatic cancer, which has been explored in our previous work and will be used to illustrate the causal cPROP framework studied in this paper [35].

### Pancreatic Cancer

**Figure 4:**
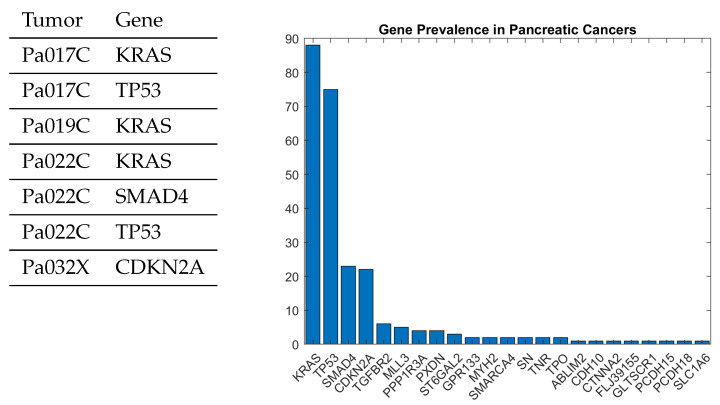
A small fragment of a dataset for analyzing pancreatic cancer [33]. Each tumor is characterized by a set of mutations of genes. The histogram plots the prevalence of each gene over all tumors in the dataset, showing that mutations in the genes KRAS and TP53 are highly indicative of pancreatic cancer.

**Figure 5:**
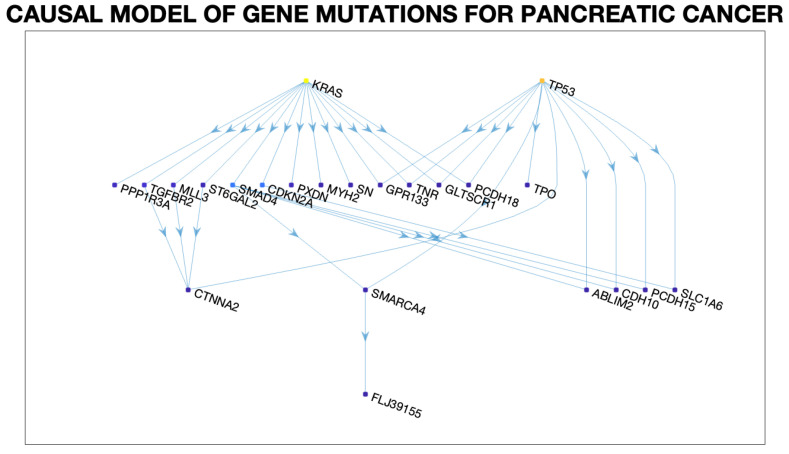
A causal DAG model of pathways in pancreatic cancer learned from a real-world dataset of around 19,000 genes and 40 tumors [33], showing genetic mutations occur along distinct pathways. The importance of mutations in the genes KRAS and TP53 is consistent with the histogram profile shown earlier in Figure 4, and the directed edges reveal possible causal pathways. To limit the size of this DAG for illustrative purposes, the model was learned only on a portion of the most widely prevalent genes that are mutated in pancreatic cancer. We are also suppressing quantitative information regarding the frequency of each gene mutation, which serves to define the model parameters (further examples are given in [38]). Such causal models can be learned through a variety of methods explored in the literature [34,38]. This particular DAG was constructed using a topological causal discovery method described in our previous work [35], building on the idea of using separating sets [6].

Figure 4 shows a small fragment of a dataset for pancreatic cancer of around 19,000 genes that are subject to mutation in around 40 tumors [33]. In any given tumor, only a relatively small number of genes are mutated. Figure 5 shows a causal DAG model learned from this dataset using a causal discovery algorithm described in greater detail in my previous work [35] based on ideas developed in [6,34]. Specifically, a causal model for pancreatic cancer can be viewed as an example of a cPROP category [16]. The pancreatic cancer causal DAG shown in Figure 5 can be straightforwardly mapped into a cPROP model using the illustrations given previously for simpler DAG models.

Like many cancers, pancreatic cancer is marked by a particular partial ordering of mutations in some specific genes, such as KRAS, TP53, and so on. In order to understand how to model and treat this deadly disease, it is crucial to understand the inherent partial ordering in the mutations of such genes. Pancreatic cancer remains one of the most prevalent and deadly forms of cancer. Roughly half a million humans contract the disease each year, most of whom succumb to it within a few years. Figure 4 shows the roughly 20 most common genes that undergo mutations during the progression of this disease. The most common gene, the KRAS gene, provides instructions for making a protein called K-Ras that is part of a signaling pathway known as the RAS/MAPK pathway. The protein relays signals from outside the cell to the cell’s nucleus. The second most common mutation occurs in the TP53 gene, which makes the p53 protein that normally acts as the supervisor in the cell as the body tries to repair damaged DNA. Like many cancers, pancreatic cancers occur as the normal reproductive machinery of the body is taken over by the cancer.

In the pancreatic cancer problem, the table in Figure 4 shows that each tumor is characterized by significant mutation events that mark the progression of the disease. In particular, the table shows that specific genes are mutated at specific locations by the change of an amino acid, causing the gene to malfunction. We can model a tumor in terms of its *genotype*, namely, the subset of *X*—the gene events that characterize the tumor. For example, the table shows that the tumor Pa022C can be characterized by the genotype KRAS, SMAD4, and TP53. We can build a causal model based on analyzing the elements of the space of genetic events and the subspaces (i.e., the genomes) that underlie the model.

The progression of many types of cancer is marked by mutations of key genes whose normal reproductive machinery is subverted by the cancer [33]. Often, viruses such as HIV and COVID-19 are constantly mutating to combat the pressure of interventions such as drugs, and successful treatment requires understanding the partial ordering of mutations. A number of past approaches use topological separability constraints on the data, assuming observed genotypes as separate events which, as will be shown, are abstractly a separability constraint on the underlying topological space.

**Figure 6:**
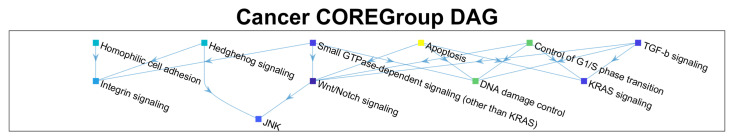
Causal DAG model of the possible 12 pathways and processes whose component genes are most likely mutated in most pancreatic cancers [33]. The specific method used to construct this causal model are described in detail in the previous work on causal homotopy [35], and related methods are described in other previous work [6,34].

A key computational level in making model discovery tractable in evolutionary processes, such as pancreatic cancer, is that multiple sources of information are available that guide the discovery of the underlying poset model. In particular, for pancreatic cancer [33], in addition to the tumor genotype information show in Figure 4, it is also known that the disease follows certain pathways, as shown in Table 2. This type of information from multiple sources gives the ability to construct multiple posets that reflect different event constraints [39]. In the previous work [35], a generalization of past algorithms that infer conjunctive Bayesian networks (CBNs) from a dataset of events (e.g., tumors or signaling pathways) and their associated genotypes (e.g., sets of genes) was described [34,39]. The causal pathways DAG shown in Figure 6 were learned using the pancreatic cancer dataset published in [33].

## 3. Greedy Equivalence Search

To motivate the theoretical development in subsequent sections, we focus our attention in this section to a specific causal discovery algorithm, Greedy Equivalence Search (GES), originally proposed by Meek [12], whose correctness and asymptotic optimality were subsequently shown by Chickering [3], constituting an a algorithmic proof of the Meek–Chickering theorem. This framework is not presented as a state-of-the-art causal discovery algorithm (e.g., Zanga and Stella [4] provide a detailed survey of many causal discovery methods), but rather as an exemplar of the idea of searching in a space of equivalence classes of DAG models. The ultimate goal is to provide a topological and abstract homotopic characterization of the search space in causal discovery, both for DAG and non-DAG models. It would help to concretize the following theoretical abstractions to ground out the ideas in a specific algorithm. The notion of a *covered edge* is fundamental to the work on causal equivalence classes in [3,12].

**Definition** **1.** *Let G=(V,E) be any causal DAG model. An edge X→Y∈E is covered if X and Y have identical parents, with the caveat that X is not a parent of itself. In other words, the parents of Y in G are the parents of X along with X itself.*

**Figure 7:**
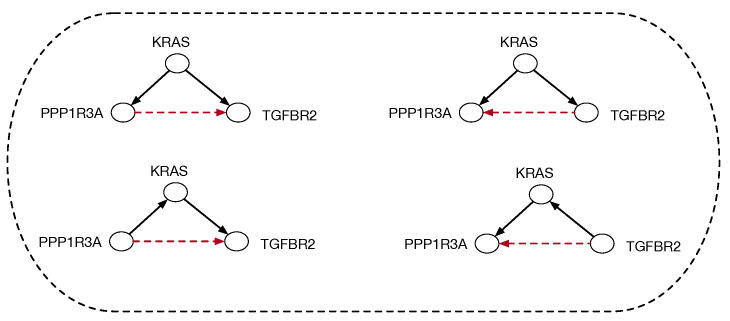
The equivalence class of DAGs that results from a single edge addition to the DAGs in Figure 2.

**Figure 8:**
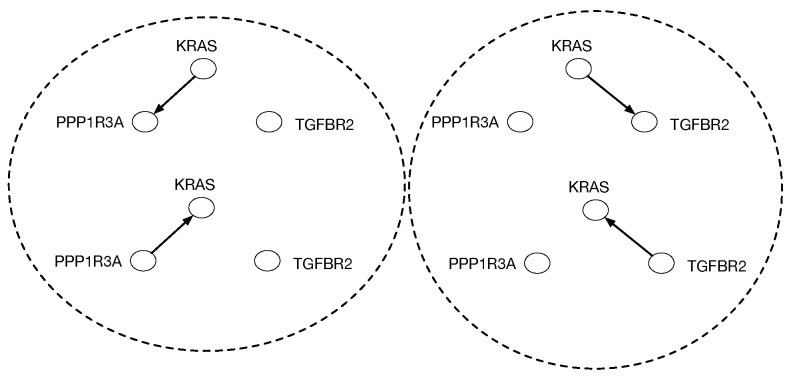
A single edge deletion to the DAGs in Figure 2 yields two equivalence classes of DAGs.

For the sake of space, the discussion of GES will be brief, and we shall relegate all missing details to the original paper [3]. Broadly, the idea underlying GES is to search over equivalence classes of DAGs by moving at each step to a *neighbor*—meaning a model outside the current equivalence class by edge addition or deletion—that has the highest Bayesian score on a given IID dataset if it improves the score. GESs in the space of DAGs that result from adding one edge to a given equivalence class in the forward direction (see Figure 7). Similarly, Figure 8 shows the two equivalence classes of DAGs that result from *deleting* a single edge to the DAGs in Figure 2. This describes the second reverse phase of the GES. It is proven in [3] that this two-phase procedure is asymptotically optimal in the limit of large datasets, provided the data were generated from some DAG. The challenge addressed in this paper is how to mathematically model the equivalence classes used in a method like GES by mapping them into equivalence among topological embeddings of string diagrams. Our motivation is to see if these ideas can be generalized to a larger class of causal models than DAGs and ultimately design improved methods, although that goal lies beyond the scope of this paper.

Bayesian approaches to learning models from data use a scoring function, such as the Bayesian Information Criterion (BIC), denoted as S(G,D), where **D** is an IID (independent and identically distributed) dataset sampled from the original (unknown) model. It is commonly assumed that such a score is locally decomposable, meaning that

$$S\left(G,D\right)=\sum_{i=1}^{n}s\left(X_{i},{\mathrm{Pa}}_{i}^{G}\right)$$

The overall score of a candidate DAG G is the sum of local scores for each node $X_{i}$ that is purely a function of the projected data **D** onto the node and its parents ${\mathrm{Pa}}_{i}^{G}$. Given a DAG G and a probability distribution p(.), G is a *perfect* map of *p* if (i) every independence constraint in *p* is implied by the structure of G and (ii) every independence constraint implied by the structure of G holds in *p*. If there exists a DAG G that is a perfect map of distribution p(.), *p* is called *DAG-perfect*. Under the assumption that the dataset **D** is an IID sample from some DAG-perfect distribution p(.), the GES algorithm consists of two phases that are guaranteed to find the correct DAG G optimally in the limit of large datasets. The precise statement is as follows.

**Theorem** **1** ([3])**.** Let $E^{*}$ denote the equivalence class that is a perfect map of the generative distribution p(.), and let m be the number of samples in a dataset **D**. Then in the limit of large m, $S_{B}\left(E^{*},D\right)>S_{B}\left(E,D\right)$ for any equivalence class $E\neq E^{*}$.

Here, $S_{B}$ is a Bayesian scoring method, like the BIC, and it is assumed to score all DAGs in an equivalence class the same. The notion of equivalence classes is obviously fundamental to the GES, and the formal statement of this characterization comes from the following transformational characterization of Bayesian networks. As previously noted, a *covered edge* in a DAG G is an edge X→Y with the property that the parents of *Y* are the same as the parents of *X* along with *X* itself.

**Theorem** **2** (**Meek–Chickering Theorem** [3,12])**.** *Let G and H be any pair of DAGs such that G≤H, meaning that H is an independence map of G, that is, every independence property in H holds in G. Intuitively, G≤H implies that H contains more edges than G. Let r be the number of edges in H that have opposite orientations in G, and let m be the number of edges in H that do not exist in either orientation in G. Then, there is a sequence of r+2m edge reversals and additions in G with the following properties:*

- *Each edge reversed is a covered edge.*
- *After each reversal and addition, G is a DAG, and G≤H.*
- *After all reversals and additions, G=H.*

To relate this result and the ensuing GES algorithm to the original PC algorithm illustrated in Figure 3, unlike the PC, the GES begins at the opposite end of the lattice of DAG models shown in Figure 2, the empty DAG (which can be viewed as the G DAG in Theorem 2), then progressively adds edges in the first phase, and then deletes edges in the second phase. In Section 9, this theorem will be generalized to construct a topological and abstract homotopical equivalence across functors between cPROP categories. These functors are equivalent to the CDU functors proposed by Jacobs et al. [9] to model Bayesian networks previously. Edge reversals or additions will correspond to natural transformations.

A further characterization of causal equivalence classes emerges from our application of higher algebraic K-theory [28,31]. Informally, we can define the notion of connectedness of a category in terms of the equivalence class of the relation defined over morphisms (two objects are in the same equivalence class if they are connected by a (perhaps zig-zag) morphism). We can treat each equivalence class as a topologically locally connected space, and then the homotopy groups $\pi_{n}\left(BC\right)$ of the classifying space BC of cPROP category C gives us an algebraic invariant of causal equivalence classes.

### Exploiting Additional Constraints in Causal Discovery

The basic idea behind the GES is to search in the space of causal equivalence classes and use a Bayesian scoring function to find the most plausible model. One significant challenge in applying the GES to the cancer domain described in Section 2 is that the datasets available are of limited size (∼100 tumor samples) but feature high-dimensionality (∼19,000 genes). The theoretical result stated in Theorem 1 may be of limited use in such situations. The approach that is generally taken in actual real-world applications of causal inference (e.g., [38]) is to bring in additional structural constraints that are motivated from particular domains. Some of these are briefly described below:

1. *Domain constraints:* In cancer, genes mutate in particular sequences, and once a gene has mutated, then it stays mutated. In other words, rather than search over all possible DAG models, it may suffice to consider restricted subclasses of DAGs, such as *conjunctive Bayesian networks* [34] (these are also referred to as “noisy-AND” models in [40]). It is often necessary to bring in such domain constraints in most real-world applications. This strategy was studied in our previous work [35] and applied to the pancreatic cancer domain.
2. *Topological representations of causal DAG models:* It is possible to convert a DAG model—viewed as a partially ordered set—into a finite space topology [41,42] by using the Alexandroff topology. In simple terms, each variable in the model is associated with its *downset* (all variables that it dominates in the partial ordering) or *upset* (all variables that dominate it in the partial ordering). The intersection of all such open or closed sets defines the Alexandroff topology embedding for each variable. This transformation can be used to determine whether two DAG (poset) models are *homotopic* and used to produce a more scalable way to enumerate posets. The results in [35,41] show several orders of magnitude improvement, at least for relatively small models.
3. *Asymptotic combinatorics:* It is a classic result from extremal combinatorics [43] that almost all partial orders of comprised of just three levels. This result is initially surprising, but the intuition behind this combinatorial result is that by carefully counting the set of all possible partial orders on *N* variables, it can be shown that as N→∞, there is a concentration phenomena that occurs where almost all partial orders are of height 3. An intriguing physical explanation is given in [44] based on phase transitions. The ramifications of this concentration phenomena were explored in a previous paper on asymptotic causality [45].

To summarize, while we will focus in the remainder of the paper on characterizing the equivalence classes of causal models using categorical techniques, it is important to point out that real-world applications will invariably require bringing in other sources of knowledge. We will return to discuss this point in Section 13.

## 4. Introduction to cPROPs and Symmetric Monoidal Categories

In this section, we will define cPROPs more formally, building on the work of Fox [18] who studied functor categories mapping PROPs to symmetric monoidal categories in his PhD dissertation in 1976. A cPROP is a functor category whose objects are functors mapping a PROP *P*—a symmetric monoidal category over natural numbers—to a symmetric monoidal category C. In the next Section 5, we will consider cPROPs where the PROP *P* is generated by a *coalgebraic* structure defined by the maps δ:1→2 and ϵ:1→0 satisfying a set of commutative diagrams. Such cPROPs are related to symmetric monoidal category structures used in previous work on categorical models of causality, probability, and statistics [10,14,23,24,25].

### 4.1. Categories and Functors

The concept of *functor* is central to category theory [16] and to our formalization of causal equivalence classes. Functors map from one category into another (possibly the same category, in which case they are viewed as *endofunctors*). Formally, a *category* C is a collection of *objects*, usually denoted by lower case letters such as c∈C and, crucially, also consisting of a collection of *morphisms* C(c,d) mapping object *c* to object *d*. Categories can be arbitrarily complex: in fact, the category **Cat** of all categories is also a category (!), which is certainly not the case for the set of all sets. In **Cat**, the set of morphisms between two categories C and D are *functors*.

Functors in general are much more expressive than functions [16]. A functor F:C→D from a domain category C to a codomain category D consists of two components:

1. An *object function* that maps each object in the domain category to an object of the codomain category. Thus, for the given functor F:C→D, for any object c∈C, the functor maps *c* to the codomain object Fc∈D. In this sense, functors resemble functions.
2. A *mapping of morphisms* that maps each morphism $f:c\to c^{\prime }$ in category C to a corresponding morphism $Ff:Fc\to Fc^{\prime }$ in category D.

cPROPs are categories comprising functor objects, as they are intended to serve as a categorical representation of Bayesian networks, structural causal models, and other types of topological causal representations studied in the literature [35,46]. Causal models map from a “syntactic” category of “diagrams” to another suitable “semantic” category. In the canonical setting of Bayesian networks that is extensively studied in our paper, the syntax category is a symmetric monoidal category that represents the structure of a DAG model (or a structural causal model (SCM) [2] or a general directed graph model [47]); the semantic category is the symmetric monoidal category **FinStoch** of stochastic processes, which represents the parameters of a Bayesian network.

### 4.2. Algebraic Theories

In an influential paper, Lawvere [20] defined an *algebraic theory* as a small category A, whose objects are the natural numbers 0,1,…, in which each object *n* is the categorical product (i.e., addition) of the unit object 1 with itself *n* times. Morphisms in A are defined as maps n→m. Lawvere [20] showed that many common algebraic structures such as groups, monoids, and rings, which are defined using finitary operations, determine an algebraic theory. Homomorphisms between algebraic structures, such as groups or rings, in turn can be used to define a category.

**Definition** **2** ([20])**.** *Every map of algebraic theories f:A→B determines a contravariant set-valued functor ${\mathrm{Sets}}^{f}:{\mathrm{Sets}}^{B}\to {\mathrm{Sets}}^{A}$, where ${\mathrm{Set}}^{f}$ is defined as an algebraic functor, and ${\mathrm{Sets}}^{A}$ is defined as an algebraic category.*

**Example** **1.** *The category of rings (with a unit element) and that of monoids are algebraic categories, and the functor that assigns to a ring the monoid consisting of the same objects under multiplication only is an algebraic functor.*

A fundamental theorem shown by Lawvere [20] states the following.

**Theorem** **3** ([20])**.** *Every algebraic functor has an adjoint.*

In terms of Example 1, the adjoint of the algebraic functor mapping rings to monoids is the free ring constructed from the elements of the monoid.

We show below that cPROPs are exactly (co)algebraic theories in the sense of Lawvere [20], as they are defined as the right adjoint of the inclusion functor from the category **CART** of all Cartesian categories into **MON**, the category of all symmetric monoidal categories. We will review these notions first before introducing cPROPs more formally.

### 4.3. Symmetric Monoidal Categories

**Figure 9:**
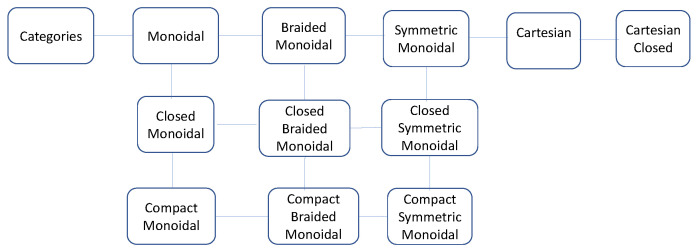
Structure of monoidal categories.

It is assumed that the reader understands the basics of symmetric monoidal categories, which are briefly reviewed below (see Figure 9). Good introductions are available in a number of textbooks [16,29]. A brief introduction to some basic category theory suitable for causal inference is found in my previous paper [14]. Detailed overviews of symmetric monoidal categories appear in many books, and the definitions presented here are based on [29].

**Definition** **3.** *A monoidal category is a category C together with a functor ⊗:C×C→C, an identity object e of C, and natural isomorphisms α,λ,ρ defined as follows:*

$$\begin{array}{ccc} \alpha_{C_{1},C_{2},C_{3}}:C_{1}⊗\left(C_{2}⊗C_{3}\right) & ≅ & \left(C_{1}⊗C_{2}\right)⊗C_{2},\;\;for\;all\;objects\;\;C_{1},C_{2},C_{3}\\ \lambda_{C}:e⊗C & ≅ & C,\;\;for\;all\;objects\;\;C\\ \rho :C⊗e & ≅ & C,\;\;for\;all\;objects\;C \end{array}$$

The natural isomorphisms must satisfy coherence conditions called the “pentagon” and “triangle” diagrams [16]. An important result shown in [16] is that these coherence conditions guarantee that all well-formed diagrams must commute.

There are many natural examples of monoidal categories, with the simplest one being the category of finite sets, termed **FinSet** in [17], where each object *C* is a set, and the tensor product ⊗ is the Cartesian product of sets, with functions acting as arrows. Deterministic causal models can be formulated in the category **FinSet**. Other examples include the category of sets with relations as morphisms and the category of Hilbert spaces [32]. The category **FinSet** has other properties, principally that the ⊗ is actually a product (in that it satisfies the universal property of products in categories and is formally a limit). Not all monoidal categories satisfy this property. Sets are also Cartesian closed categories, meaning that there is a right adjoint to the tensor product, which represents exponential objects and is often referred to as the “internal hom” object. Markov categories to be defined in Section 5 are monoidal categories, where the identity element *e* is also a terminal object, meaning there is a unique “delete” morphism $d_{e}:X\to e$ associated with each object *X*. This property can be used to show that projections of tensor products X⊗Y exist, but they do not satisfy the universal property. We will return to this question below in Section 5.1. Markov categories do not satisfy uniform copying.

**Definition** **4.** *A symmetric monoidal category is a monoidal category (C,⊗,e,α,λ,ρ), together with a natural isomorphism*

$$\begin{array}{c} \tau_{C_{1},C_{2}}:C_{1}⊗C_{2}≅C_{2}⊗C_{1},\;\;for\;all\;objects\;\;C_{1},C_{2} \end{array}$$

*where τ satisfies the additional conditions: for all objects $C_{1},C_{2}$$\tau_{C_{2},C_{1}}\circ \tau_{C_{1},C_{2}}≅1_{C_{1}⊗C_{2}}$ and for all objects C, $\rho_{C}=\lambda_{C}\circ \tau_{C,e}:C⊗e≅C$.*

An additional hexagon axiom is required to ensure that the τ natural isomorphism is compatible with α. The τ operator is called a “swap” in Markov categories [17]. These isomorphisms are easier to visualize as string diagrams, as will be illustrated below in Section 5.

### 4.4. PROPs

The structure PROP (for Products and Permutations) was originally introduced by Maclane [15], and it has seen widespread use in many areas such as modeling connectivity in networks [21,22]. A trivial example of a PROP is the free monoidal category Γ over the category 1, whose objects can be interpreted as the natural numbers, the unit object 0, and the tensor product being addition. More generally, a PROP *P* is a small monoidal category with a strict monoidal functor Γ→P that is a bijection on objects. A cPROP is a functor category $C^{P}$, where C is a symmetric monoidal category, and in addition, there are usually some constraints placed on the specific PROP *P*.

**Definition** **5** ([18])**.** *A PROP $\underline{P}$ is a small symmetric monoidal category with a strict monoidal functor $\Gamma \to \underline{P}$, which is a bijection on objects. A PROP $\underline{P}$ is algebraic (respectively, coalgebraic) if its set of maps is generated by a set of maps having codomain 1 (respectively, domain 1).*

Given a map σ in $\underline{P}$, let σd and σr define its domain and range, respectively (both are natural numbers). Fox [18] defines a *propable* category $C^{P}$ as one that satisfies the following commutative diagram:

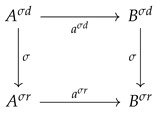


For example, given the PROP map ϵ:1→0, the commutative diagram states that there is a natural transformation $\epsilon_{X}:X\to I$ for each object *X* in C (where $X^{0}=I$ and is the unit element). In Section 5, we will see that this structure defines the ${\mathrm{del}}_{X}$ “delete” structure in Markov and affine CDU categories.

### 4.5. cPROPs

We now define a cPROP as a functor category whose objects are functors from a PROP $\underline{P}$ to a symmetric monoidal category C.

**Definition** **6.** *A cPROP is a functor category from a PROP $\underline{P}$ to a symmetric monoidal category C, which comes with the usual forgetful functor mapping a functor in cPROP to a symmetric monoidal category C.*

We are usually interested in cPROPs with a special structure on $\underline{P}$, which captures some of the typical structures used in applications, such as causal inference [1,2]. In such cases, we would like to be able to represent probability distributions, do causal interventions on graphs representing distributions, and marginalize over distributions to compute answers using rules like those of do-calculus [2]. We identify one simple example of such a regularity, which will turn out to be important in terms of its relationship to previous work on categorical causal models discussed in Section 5.

**Definition** **7.** *Let $c\underline{P}$ denote a cPROP as a functor category from a coalgebraic PROP $\underline{P}$ generated by the maps δ:1→2 and ϵ:1→0, satisfying the following commutative diagrams (where τ is a “twist” morphism also commonly referred to as a braiding [16]).*

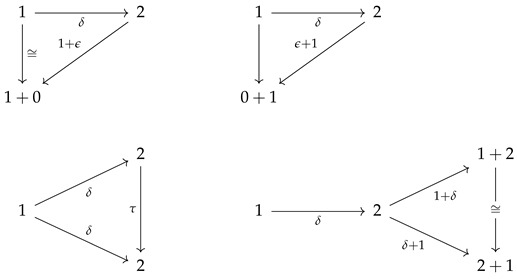




Note that the δ coalgebraic map in Markov and affine CDU categories defines the ${\mathrm{copy}}_{X}$ map, which we discuss in more detail in Section 5. Fox [18] shows that the cPROP category $c\underline{P}$ of coalgebraic structures defined by the above commutative diagrams is Cartesian. This result also is shown by Heunen and Vicary [32], whose work is discussed in Section 5. It is worth emphasizing that in Markov categories, ${\mathrm{del}}_{X}$ is assumed to obey its commutative diagram above, but ${\mathrm{copy}}_{X}$ does not. We can easily model a Markov category as a cPROP where the commutative diagram for copy is not imposed uniformly over the category C as it is for del.

**Theorem** **4** ([18])**.** *The cPROP category $c\underline{P}$ is Cartesian.*

**Proof.**  The category $c\underline{P}$ consists of comonoidal objects $\left(C_{i},\delta_{i},\epsilon_{i}\right)$ over which a tensor product structure can be defined as follows. If $\left(C_{1},\epsilon_{1},\delta_{1}\right)$ and $\left(C_{2},\delta_{2},\epsilon_{2}\right)$ are two objects in $c\underline{P}$, their tensor product in $c\underline{P}$ is defined to be object

$$\left(C_{1}⊗C_{2},\left(1⊗\tau ⊗1\right)\circ \left(\delta_{1}⊗\delta_{2}\right),\epsilon_{1}⊗\epsilon_{2}\right)$$

It may be easier to visualize this as a string diagram (see Equation (4) for the specific example from Markov categories). To show that this particular tensor product is actually the categorical product in $c\underline{P}$, let (C,δ,ϵ) be any object in $c\underline{P}$, and let the “projection” arrows in $c\underline{P}$ be defined as $a:C\to C_{1}$ and $b:C\to C_{2}$. A diagram chase using the below commutative diagram shows that $1⊗\epsilon_{2}:C_{1}⊗C_{2}\to C_{1}$ is indeed the product projection.

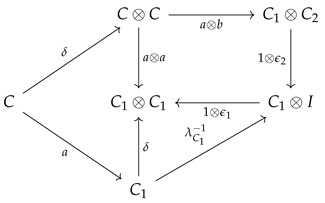

   □

Fox [18] additionally proved the following result, showing that the coalgebraic category $c\underline{P}$ forms a Cartesian closure of the category of symmetric monoidal categories **MON**.

**Theorem** **5** ([18])**.** *Define* **CART** *to be the category of all Cartesian categories with strictly product-preserving functors and* **MON** *to be the category of symmetric monoidal categories with strict monoidal functors as arrows. Then, the functor F that maps a category in* **MON** *to* **CART** *via its coalgebraic PROP structure is right adjoint to the inclusion functor from* **CART** *to* **MON**.

**Proof.**  Define a functor *F* that maps a category in **MON** to the CPROP category $c\underline{P}$ using the PROP maps δ:1→2 and ϵ:1→0. Let D be any Cartesian category, and let F:D→C be an arrow in **MON**, i.e., a strictly monoidal functor. For any object *X* in D, let Δ:X→X×X be its diagonal map (which exists because D is Cartesian), and define the composed morphism $\epsilon_{X}:X\overset{≅}{\to }X⊗I\overset{\pi }{\to }I$ (where the projection π exists because *I* is terminal). Define the functor *F* that maps from **MON** to **CART** by mapping an object *X* to the comonoidal object $c\underline{P}$ as (F(X),F(Δ),F(ϵ)). *F* preserves products by the diagram chase shown above. Then, *F* has a left adjoint defined by the forgetful functor *U* from the cPROP category $c\underline{P}$ to **MON**.    □

### 4.6. Closed Locally Presentable cPROPs

We turn now to discuss the property of closedness and accessibility in cPROP categories. These will be useful in forming exponential objects, as well as in being able to apply the special adjoint functor theorem (SAFT) [16] (Theorem 2, Section V.8).

**Definition** **8.** *A cPROP category C is closed Cartesian if it has all finite products and if the symmetric monoidal structure (C,×,e) is closed. In other words, for all objects C, the functor (−)⊗C possess a right adjoint (which is referred to as an “exponential” object or an “internal hom” object).*

$$C\left(X⊗Y,Z\right)≅C\left(X,Z^{Y}\right)$$

We define subobjects of the objects in cPROP categories.

**Definition** **9.** *For any cPROP category C, given two monomorphisms f:X→Y and g:Z→Y that share a common co-domain, let f≤g when f factors through g, namely, $f=g\circ f^{\prime }$ for some arrow $f^{\prime }$ (which must also be a monomorphism). If both f≤g and g≤f, the induced equivalence classes of monomorphisms f≡g with codomain Y define the subobjects of Y.*

To construct accessible cPROP categories, they need to be locally presentable through a cogenerating set M of objects.

**Definition** **10.** *A cogenerating set of objects M for a cPROP category C that exists if for every parallel pair of arrows $h\neq h^{\prime }:X\to Y$, there is an object Q in M and an arrow g:Y→Q such that $g\circ h\neq g\circ h^{\prime }$.*

This property lets us construct initial objects for cPROP categories.

**Theorem** **6** ([16])**.** *Special Initial-Object Theorem: If a cPROP category C is small-complete (implying that it has finite products and a terminal object), and there is a small cogenerating set M, then C has an initial object assuming every set of subobjects of X in C has a finite intersection.*

The proof is simple and involves constructing the product $X_{0}$ of all objects in the cogenerating set M and then taking the intersection of all the subobjects of $X_{0}$. Since the set of subobjects is a partial ordering under the relation ≤ in Definition 9, and the Cartesianness of the cPROP category gives us pullbacks, we can use this universal construction to find the meet or intersection of any set of subobjects. An important result that depends on this property is the Special Adjoint Functor Theorem [16].

**Theorem** **7.** *Special Adjoint Functor Theorem (SAFT): Given a small-complete cPROP category M, with small hom sets and a small cogenerating set M, where every set of subobjects of objects in C has a pullback, then any functor G:C→D has a left adjoint if and only if G preserves all small limits and all pullbacks of families of monomorphisms.*

Fox [18] showed that the cPROP category $c\underline{P}$ has a small cogenerating set of objects and that the functor *F* from $c\underline{P}$ to **MON** creates colimits. This was used to show that $c\underline{P}$ is locally presentable, and by the SAFT, there exists a right adjoint to the forgetful functor $U:c\underline{P}\to \mathrm{MON}$.

## 5. Affine CDU and Markov Categories as cPROPs

In this section, we will relate cPROPs to previous work on affine CDU categories [25] and Markov categories [17]. Markov categories have been studied extensively as a unifying categorical model for causal inference, probability, and statistics. They are symmetric monoidal categories, which we reviewed in Section 4.3, combined with a comonoidal structure on each object. Importantly, Markov categories are semi-Cartesian because they do not use uniform copying but contain a Cartesian subcategory defined by deterministic morphisms. I give a brief review of Markov categories, and significant additional details that are omitted can be found in [10,17,25]. For the sake of clarity, we will follow the definitions in [17], although we will explore some of the subtleties in these definitions in Section 5.1 relating to the Cartesian structure of a Markov category.

**Definition** **11.** *A* Markov category *C [17] is a symmetric monoidal category in which every object X∈C is equipped with a commutative comonoid structure given by a comultiplication ${\mathrm{copy}}_{X}:X\to X⊗X$ and a counit ${\mathrm{del}}_{X}:X\to I$, depicted in string diagrams as*

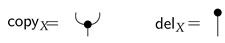

(1)

*and satisfying the commutative comonoid equations*

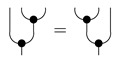

(2)



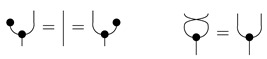

(3)

*as well as compatibility with the monoidal structure*

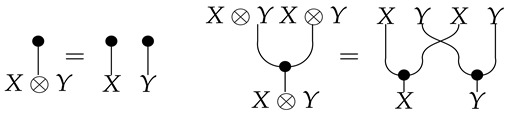

(4)

*and the naturality of del, which means that*

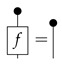

(5)

*for every morphism f.*

Note that to adequately represent discovery algorithms like PC, and their many extensions and variants like Greedy Equivalent Search [3], it is necessary to modify string diagrams to represent equivalence class of causal models. The challenge we have to face is that causal discovery requires searching through a super-exponentially large space of such string diagrams. Note that string diagrams defined over Markov categories are essentially induced by the PROP maps that define their (co)algebraic structure. In Section 7 and Section 8, it will be shown how such string diagrams can be converted into continuous maps over “nice” topological spaces, in particular CW-complexes using the nerve functor that maps a (symmetric monoidal) category into a simplicial set [28]. Thus, the tensor product bifunctor ⊗:C×C→C leads to an *H*-space, or a topological space with a chosen basepoint (which can be defined as the topological 0-cell associated with the terminal object *I* in a Markov category), as well as a continuous map BC×BC→BC. The comonoid comultiplication ${\mathrm{copy}}_{X}:X\to X⊗X$ induces a diagonal map BC→BC×BC. Since *I* is a terminal object in a Markov category, its classifying spaces BC are contractible.

**Figure 10:**
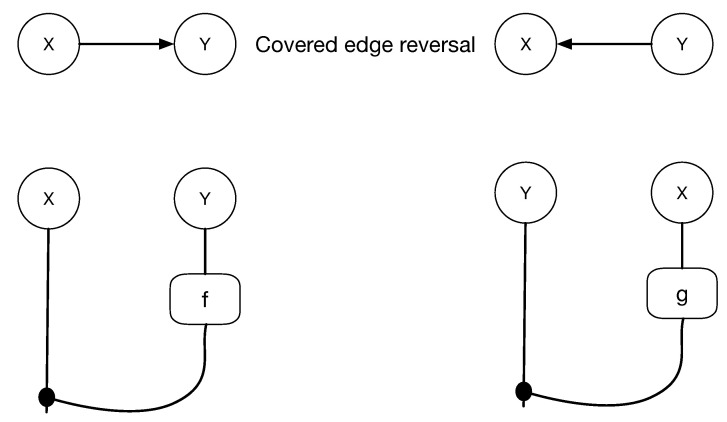
Meek–Chickering equivalence by reversal of covered edges induces an equivalence of the associated string diagrams.

Figure 10 illustrates a cPROP Markov category string diagram showing how the reversal of covered edges induces an equivalence of the associated string diagrams.

### 5.1. Cartesian Structure in Markov Categories

I now discuss a subcategory of Cartesian categories within Markov that involves uniform ${\mathrm{copy}}_{X}$ and ${\mathrm{del}}_{X}$ morphisms. One fundamental property of Markov categories is that they are *semi-Cartesian*, as the unit object is also a terminal object. But, a subtlety arises in how these copy and delete operators are modeled, as will be discussed below.

**Definition** **12.** *A symmetric monoidal category C is Cartesian if the tensor product* ⊗ *is the categorical product*.

If C and D are symmetric monoidal categories, then a functor F:C→D is monoidal if the tensor product is preserved up to coherent natural isomorphisms. *F* is strictly monoidal if all the monoidal structures are preserved exactly, including ⊗, unit object *I*, symmetry, and associative and unit natural isomorphisms. Denote the category of symmetric monoidal categories with strict functors as arrows as **MON**. Let us review the basic definitions given by Heunen and Vicary [32], which will give some further clarity on the Cartesian structure in affine CDU and Markov categories.

**Definition** **13.** *The subcategory of comonoids* **coMON** *in the ambient category* **MON** *of all symmetric monoidal categories is defined for any specific category C as a collection of “coalgebraic” objects $\left(X,{copy}_{X},{del}_{X}\right)$, where X is in C, and arrows defined as comonoid homomorphisms from $\left(X,{\mathrm{copy}}_{X},{\mathrm{del}}_{X}\right)$ to $\left(Y,{\mathrm{copy}}_{Y},{\mathrm{del}}_{Y}\right)$ act uniformly in the sense that if f:X→Y is any morphism in C, then*

$$\begin{array}{c} \left(f⊗f\right)\circ {\mathrm{copy}}_{X}={\mathrm{copy}}_{Y}\circ f\\ {\mathrm{del}}_{Y}\circ f={\mathrm{del}}_{X} \end{array}$$

Heunen and Vicary [32] defined the process of “uniform copying and deleting” in the category **coMON**, which we will now relate to Markov categories. A subtle difference worth emphasizing with Definition 11 is that in Markov categories, only ${\mathrm{del}}_{X}$ is “uniform”, but not ${\mathrm{copy}}_{X}$ in the sense defined by Heunen and Vicary [32]. This distinction can be modeled in a cPROP category that is semi-Cartesian like Markov categories by suitably modifying the definition of the associated PROP map for copying.

**Definition** **14** ([32])**.** *A symmetric monoidal category C admits uniform deleting if there is a natural transformation $e_{X}:X\overset{e_{X}}{\to }I$ for all objects in the subcategory $C_{\mathrm{coMON}}$ of comonoidal objects, where $e_{I}={id}_{I}$, as shown in Equation (5).*

This condition was referred to by Cho and Jacobs [25] as a *causality* condition on the arrow $e_{X}$. Essentially, it states that if you process some object and then discard it, it is equivalent to discarding it without processing.

**Theorem** **8** ([32])**.** *A symmetric monoidal category C has uniform deleting if and only if I is terminal.*

This property holds for Markov categories, as noted in [17], and a simple diagram chasing proof is given in [32].

**Definition** **15** ([32])**.** *A symmetric monoidal category C has uniform copying if there is a natural transformation ${\mathrm{copy}}_{X}:X\to X⊗X$ such that ${\mathrm{del}}_{I}=\rho_{I}^{-1}$, satisfying Equations (2) and (3).*

We can now state an important result proved in [32] (Theorem 4.28), which relates to the more general results shown earlier by Fox [18]

**Theorem** **9** ([18,32])**.** *The following conditions are equivalent for a symmetric monoidal category C:*

- *The category C is Cartesian, with tensor products* ⊗ *given by the categorical product, and the tensor unit is given by the terminal object.*
- *The symmetric monoidal category C has uniform copying and deleting, and Equation (2) holds.*

As noted by Fritz [17], not all Markov categories are Cartesian, because their ${\mathrm{copy}}_{X}$ is not uniform, but only ${\mathrm{del}}_{X}$ is. For example, consider the category **FinStoch**, where a joint distribution is specified by the morphism ψ:I→X⊗Y. In this case, the marginal distributions can be formed as the composite morphisms

$$\begin{array}{c} I\overset{\psi }{\to }X⊗Y\overset{{\mathrm{del}}_{Y}}{\to }X\\ I\overset{\psi }{\to }X⊗Y\overset{{\mathrm{del}}_{X}}{\to }Y \end{array}$$

But to require that in this case ⊗ is the categorical product implies that the marginal distributions defined as the above composites must be in bijection with the joint distribution.

### 5.2. cPROP Causal Category for Pancreatic Cancer

We can transform the causal DAGs in Figure 5 and Figure 6 into equivalent cPROP string diagram categorical models using the general process of mapping DAGs into string diagram category structures (see [9,17]). For example, for the cPROP category underlying the causal model in Figure 5, we can now construct a morphism for each edge in this DAG model, such as

f:KRAS→SMAD4,g:TP53→TPO,

and so on for the other edges. Analogously, we can construct a cPROP category based on the causal DAG pathway model in Figure 6, whose morphisms would include

f:Homophiliccelladhesion→Integrinsignalling,

and similar morphisms corresponding to other edges. An intriguing direction for further exploration of this application is to construct a symmetric monoidal product cPROP category that combines both these cPROP categories into a product cPROP category.

We can also construct equivalence classes of these two causal DAGs using reversals of covered edges (and their corresponding natural transformations mapping each causal DAG in an equivalence class into a cPROP functor object), as described in Section 8. A crucial advantage of our proposed framework in this domain is that by capturing the underlying equivalence classes more compactly using the higher-algebraic k-theory framework, it opens the possibility of constructing more compact models that include a larger percentage of the genes mutated by pancreatic cancer. Since there are potentially tens of thousands of genes, previous work was limited to building models on only the most likely mutated genes, as the number of possible DAG models grows explosively in size, as noted in the introduction. A more detailed analysis of this application using the theoretical framework proposed below will be reported in a subsequent paper and is beyond the scope of this paper.

To summarize this section, we reviewed affine CD categories [25] and Markov categories [17] and showed that they are closely related to cPROPs. Markov categories are semi-Cartesian, as the unit element *I* is terminal, but they are not Cartesian, as they do not allow uniform copying. They do contain a subcategory defined by their comonoidal objects that is Cartesian. In the remainder of this paper, we will construct simplicial objects over cPROP categories, and then in Section 8, we will define the basic concepts of homotopies in cPROP categories.

## 6. Structural Causal Models as Cartesian cPROPs

In this section, we will briefly discuss how Pearl’s structural causal models (SCMs) [2] can be defined as a special type of cPROP involving deterministic morphisms, as defined in [17]. Recall from the previous section that, in general, a Markov category has uniform deletion but not uniform copying. However, as noted in [17], a morphism is defined to be deterministic if it leads to uniform copying. Using the results from Theorem 9 above, we can conclude therefore that the subclass of Markov categories with uniform copying and deleting is Cartesian and hence sufficient to model SCMs in so far as the mapping from exogenous variables to endogenous variables is deterministic. Recall that in an SCM, the mapping from exogenous variables (i.e., variables external to the model) to endogenous variables (i.e., variables internal to the model) is purely *deterministic*. The uncertainty derives purely from the fact that external variables are assumed to be defined with respect to some probability distribution. We will begin with a review of Pearl’s SCM framework and then show how it corresponds to a special type of cPROP Markov category using deterministic morphisms.

**Definition** **16** ([2])**.** *A structural causal model (SCM) is defined as the triple 〈U,V,F〉 where $V=\{V_{1},\ldots ,V_{n}\}$ is a set of* endogenous *variables, U is a set of* exogenous *variables, and F is a set $\left\{f_{1},\ldots ,f_{n}\right\}$ of purely deterministic “local functions” $f_{i}:U\cup \left(V\setminus V_{i}\right)\to V_{i}$ whose composition induces a unique function F from U to V.*

**Definition** **17** ([2])**.** *Let M=〈U,V,F〉 be a causal model defined as an SCM, let X be a subset of variables in V, and let x be a particular realization of X. A submodel $M_{x}=\langle U,V,F_{x}\rangle$ of M is the causal model $M_{x}=\langle U,V,F_{x}\rangle$, where $F_{x}=\left\{f_{i}:V_{i}\notin X\right\}\cup \left\{X=x\right\}$.*

**Definition** **18** ([2])**.** *Let M be an SCM, X be a set of variables in V, and x be a particular realization of X. The effect of an action do(X=x) on M is given by the submodel $M_{x}$.*

**Definition** **19** ([2])**.** *Let Y be a variable in V, and let X be a subset of V. The potential outcome of Y in response to an action do(X=x), denoted $Y_{x}\left(u\right)$, is the solution of Y for the set of equations $F_{x}$.*

### SCMs as Cartesian cPROPs

Recall from Theorem 9 that symmetric monoidal categories that are Cartesian admit uniform copying and deleting. We can use this property to define deterministic morphisms in a cPROP (or Markov) category as follows.

**Definition** **20** ([17])**.** *A morphism f:X→Y in a cPROP category C is deterministic if it admits uniform copying.*

We can now define an SCM as a cPROP category where all the morphisms between exogenous variables and endogenous variables are deterministic.

**Definition** **21.** *A structural causal model (SCM) [2] can be defined as a restricted type of cPROP category $C_{\mathrm{SCM}}$ whose collection of objects Obj={U,V} is partitioned into a collection of exogenous objects U and a collection of endogenous objects V such that every morphism f:X→Y from an exogenous object X∈U to an endogenous object Y∈V is deterministic.*

Observe that any product of exogenous objects $X_{1}⊗X_{2}\ldots X_{n}$ is exogenous if each object $X_{i}$ is exogenous, and similarly, any product of endogenous objects is also endogenous if each object in the product is endogenous. Thus, every exogenous variable is defined with respect to some probability distribution *P*.

**Definition** **22.** *Given a Cartesian cPROP category $C_{\mathrm{SCM}}$ corresponding to a structural causal model, to every object corresponding to an exogenous variable X, there exists a morphism ψ:I→X that defines a distribution over X.*

Figure 1 illustrates an example of a structural causal model defining pollution in New Delhi, India (based on the original example in [14]), translated into a string diagram defining a Cartesian cPROP. Note that the exogenous variables here are defined by probability distributions (morphisms) in a Markov category as follows:

ψ:I→Overpopulation,ϕ:I→CovidLockdown,δ:I→FarmingPractices

The Cartesian model specifying the endogenous variables are defined purely using deterministic morphisms, which includes the following:

$$\begin{array}{ccc} f:\mathrm{Overpopulation} & ⊗ & \mathrm{Covid}\;\mathrm{Lockdown}\to \mathrm{Traffic}\\ g:\mathrm{Covid}\;\mathrm{Lockdown} & ⊗ & \mathrm{Farming}\;\mathrm{Practices}\to \mathrm{Agricultural}\;\mathrm{practices} \end{array}$$

and is similar for the morphisms *h* and *w*.

## 7. Simplicial Objects in cPROPs

**Figure 11:**
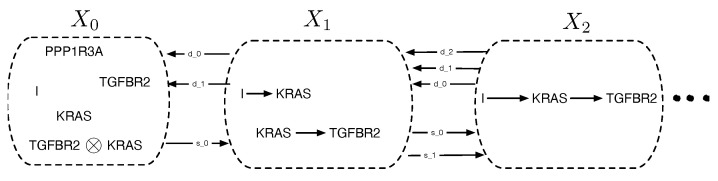
A simplicial object in a cPROP category. $X_{0}$ defines the 0-simplices represented by objects: in the pancreatic cancer domain, these represent individual mutated genes or the initial object *I* (denoting a marginalized distribution). $X_{1}$ defines the 1-simplices represented by single morphisms, such as the distribution I→KRAS. Similarly, 2-simplices represent composable morphisms of length 2, such as I→KRAS→TGFBR2 and so on.

We now turn to the embedding of cPROPs in the category of simplicial sets, which will be a prelude to constructing “nice” topological realizations and the study of their classifying spaces. Figure 11 gives the high level intuition. A simplicial set *X* is defined as a collection of sets $X_{0},X_{1},\ldots$, which is combined with face maps (indicated as $d_{i}$ in the figure) and degeneracy maps (indicated as $s_{j}$ in the figure). As a simple guide to help build intuition, any directed graph can be viewed as a simplicial set, where $X_{0}$ is the set *V* of vertices, $X_{1}$ is the *E* of edges, and the two face maps $d_{0}$ and $d_{1}$ from $X_{1}$ to $X_{0}$ yield the initial and final vertex of the edge. The single degeneracy map $s_{0}$ between $X_{0}$ and $X_{1}$ adds a self loop to each vertex. Simplicial sets generalize graphs when we consider higher-order simplices. For example, between $X_{1}$ and $X_{2}$, there are three face maps, mapping a simplicial triangle (a 2-simplex) Δ to each of its 1-simplicial components, namely, its edges.

A brief review of simplicial sets is given, summarizing some points made in my previous paper on simplicial set representations in causal inference [14]. A more detailed review can be found in many references [29,48]. Simplicial sets are higher-dimensional generalizations of directed graphs, partially ordered sets, and regular categories themselves. Importantly, simplicial sets and simplicial objects form a foundation for higher-order category theory [26,27]. Using simplicial sets and objects enables a powerful machinery to reason about both directional and non-directional paths in causal models and to model equivalence classes of causal models.

Simplicial objects have long been a foundation for algebraic topology [48,49] and more recently in higher-order category theory [26,27,50]. The category Δ has non-empty ordinals [n]={0,1,…,n] as objects and order-preserving maps [m]→[n] as arrows. An important property in Δ is that any many-to-many mapping is decomposable as a composition of an injective and a surjective mapping, each of which is decomposable into a sequence of elementary injections $\delta_{i}:\left[n\right]\to \left[n+1\right]$ called *coface* mappings, which omit i∈[n], and a sequence of elementary surjections $\sigma_{i}:\left[n\right]\to \left[n-1\right]$, called *co-degeneracy* mappings, which repeat i∈[n]. The fundamental simplex Δ([n]) is the presheaf of all morphisms into [n], that is, the representable functor Δ(−,[n]). The Yoneda Lemma [16] assures us that an *n*-simplex $x\in X_{n}$ can be identified with the corresponding map Δ[n]→X. Every morphism f:[n]→[m] in Δ is functorially mapped to the map Δ[m]→Δ[n] in S.

Any morphism in the category Δ can be defined as a sequence of *codegeneracy* and *coface* operators, where the coface operator $\delta_{i}:\left[n-1\right]\to \left[n\right],0\leq i\leq n$ is defined as

$$\delta_{i}\left(j\right)=\left\{\begin{array}{cc} j, & \mathrm{for}\;0\leq j\leq i-1\\ j+1 & \mathrm{for}\;i\leq j\leq n-1 \end{array}\right.$$

Analogously, the codegeneracy operator $\sigma_{j}:\left[n+1\right]\to \left[n\right]$ is defined as

$$\sigma_{j}\left(k\right)=\left\{\begin{array}{cc} j, & \mathrm{for}\;0\leq k\leq j\\ k-1 & \mathrm{for}\;j<k\leq n+1 \end{array}\right.$$

Note that under the contravariant mappings, coface mappings turn into face mappings, and codegeneracy mappings turn into degeneracy mappings. That is, for any simplicial object (or set) $X_{n}$, we have $X\left(\delta_{i}\right)≔d_{i}:X_{n}\to X_{n-1}$, and likewise, $X\left(\sigma_{j}\right)≔s_{j}:X_{n-1}\to X_{n}$.

The compositions of these arrows define certain well-known properties [29,48]:

$$\begin{array}{ccc} \delta_{j}\circ \delta_{i} & = & \delta_{i}\circ \delta_{j-1},\;\;i<j\\ \sigma_{j}\circ \sigma_{i} & = & \sigma_{i}\circ \sigma_{j+1},\;\;i\leq j\\ \sigma_{j}\circ \delta_{i}\left(j\right) & = & \left\{\begin{array}{cc} \sigma_{i}\circ \sigma_{j+1}, & \mathrm{for}\;i<j\\ 1_{\left[n\right]} & \mathrm{for}\;i=j,j+1\\ \sigma_{i-1}\circ \sigma_{j},\mathrm{for}\;i>j+1 \end{array}\right. \end{array}$$

**Example** **2.** *The “vertices” of a simplicial object X in a cPROP category C are the objects $X_{0}$ in C, and the “edges” $X_{1}$ are its arrows $f:C_{i}\to C_{j}$, where $C_{i}$ and $C_{j}$ are objects in C. Note that $X_{0}$ is a contravariant functor X:[0]→C, and since [0] has only one object, the effect of this functor is to pick out objects in C. The simplicial object is $X_{1}:\left[1\right]\to C$. Given any such arrow, the face operators $d_{0}f=C_{j}$ and $d_{1}f=C_{i}$ recover the source and target of each arrow. Also, given an object X of category C, we can regard the degeneracy operator $s_{0}X$ as its identity morphism $1_{X}:X\to X$.*

**Example** **3.** *Given a cPROP category C, we can identify an n-simplex $X_{n}$ of a simplicial object in a cPROP category $sC_{n}$ with the following sequence:*

$$X_{n}=C_{o}\overset{f_{1}}{\to }C_{1}\overset{f_{2}}{\to }\ldots \overset{f_{n}}{\to }C_{n}$$

*and the face operator $d_{0}$ applied to $X_{n}$ yields the sequence*

$$d_{0}X_{n}=C_{1}\overset{f_{2}}{\to }C_{2}\overset{f_{3}}{\to }\ldots \overset{f_{n}}{\to }C_{n}$$

*where the object $C_{0}$ is “deleted” along with the morphism $f_{0}$ leaving it.*

**Example** **4.** *Given a cPROP category C and an n-simplex $X_{n}$ of the simplicial object in a cPROP category $X_{n}$, the face operator $d_{n}$ applied to $X_{n}$ yields the sequence*

$$d_{n}X_{n}=C_{0}\overset{f_{1}}{\to }C_{1}\overset{f_{2}}{\to }\ldots \overset{f_{n-1}}{\to }C_{n-1}$$

*where the object $C_{n}$ is “deleted” along with the morphism $f_{n}$ entering it.*

**Example** **5.** *Given a cPROP category C and an n-simplex $X_{n}$ of the simplicial object, the face operator $d_{i},0<i<n$ applied to $X_{n}$ yields the sequence*

$$d_{i}X_{n}=C_{0}\overset{f_{1}}{\to }C_{1}\overset{f_{2}}{\to }\ldots C_{i-1}\overset{f_{i+1}\circ f_{i}}{\to }C_{i+1}\ldots \overset{f_{n}}{\to }C_{n}$$

*where the object $C_{i}$ is “deleted”, and the morphisms $f_{i}$ are composed with morphism $f_{i+1}$.*

**Example** **6.** *Given a cPROP category C and an n-simplex $X_{n}$ of the simplicial object defined over the cPROP category, the degeneracy operator $s_{i},0\leq i\leq n$ applied to $X_{n}$ yields the sequence*

$$s_{i}X_{n}=C_{0}\overset{f_{1}}{\to }C_{1}\overset{f_{2}}{\to }\ldots C_{i}\overset{1_{C_{i}}}{\to }C_{i}\overset{f_{i+1}}{\to }C_{i+1}\ldots \overset{f_{n}}{\to }C_{n}$$

*where the object $C_{i}$ is “repeated” by inserting its identity morphism $1_{C_{i}}$.*

**Definition** **23.** *Given a cPROP category C and an n-simplex $X_{n}$ of the simplicial object associated with the category, $X_{n}$ is a degenerate simplex if some $f_{i}$ values in $X_{n}$ are an identity morphism, in which case $C_{i}$ and $C_{i+1}$ are equal.*

### 7.1. Nerve of a Category

There is a general way to construct a simplicial set representation of any category by constructing its *nerve* functor [28]. This construction formalizes what was illustrated in the above examples.

**Definition** **24.** *The nerve of a category C is the set of composable morphisms of length n for n≥1. Let $N_{n}\left(C\right)$ denote the set of sequences of composable morphisms of length n.*

$$\left\{C_{o}\overset{f_{1}}{\to }C_{1}\overset{f_{2}}{\to }\ldots \overset{f_{n}}{\to }C_{n}\;|\;C_{i}\;is\;an\;object\;in\;C,f_{i}\;is\;a\;morphism\;in\;C\right\}$$

The set of *n*-tuples of composable arrows in C, denoted by $N_{n}\left(C\right)$, can be viewed as a functor from the simplicial object [n] to C. Note that any non-decreasing map α:[m]→[n] determines a map of sets $N_{m}\left(C\right)\to N_{n}\left(C\right)$. The nerve of a category C is the simplicial set $N_{•}:\Delta \to N_{n}\left(C\right)$, which maps the ordinal number object [n] to the set $N_{n}\left(C\right)$.

The importance of the nerve of a category comes from a key result [29,51], showing that it defines a full and faithful embedding of a category.

**Theorem** **10.** *The nerve functor $N_{•}:$* **Cat** *→* **Set** *is fully faithful. More specifically, there is a bijection θ defined as*

$$\theta :\mathrm{Cat}\left(C,C^{\prime }\right)\to {\mathrm{Set}}_{\Delta }(N_{•}\left(C\right),N_{•}\left(C^{\prime }\right)$$

Unfortunately, the left adjoint to the nerve functor is not a full and faithful encoding of a simplicial set back into a suitable category. Note that a functor *G* from a simplicial object *X* to a category C can be lossy. For example, we can define the objects of C to be the elements of $X_{0}$ and the morphisms of C as the elements $f\in X_{1}$, where f:a→b, $d_{0}f=a$, $d_{1}f=b$, and $s_{0}a,a\in X$ define the identity morphisms $1_{a}$. Composition in this case can be defined as the free algebra defined over elements of $X_{1}$, which is subject to the constraints given by elements of $X_{2}$. For example, if $x\in X_{2}$, we can impose the requirement that $d_{1}x=d_{0}x\circ d_{2}x$. Such a definition of the left adjoint would be quite lossy because it only preserves the structure of the simplicial object *X* up to the 2-simplices. The right adjoint from a category to its associated simplicial object, in contrast, constructs a full and faithful embedding of a category into a simplicial set. In particular, the nerve of a category is such a right adjoint.

### 7.2. Topological Embedding of cPROP Categories

Simplicial objects in cPROP categories can be embedded in a topological space using a construction originally proposed by Milnor [30].

**Definition** **25.** *The geometric realization |X| of a simplicial object X in the cPROP category C is defined as the topological space*

$$\left|X\right|=\underset{n\geq 0}{⨆}X_{n}\times \Delta^{n}/\;∼$$

*where the n-simplex $X_{n}$ is assumed to have a* discrete *topology (i.e., all subsets of $X_{n}$ are open sets), and $\Delta^{n}$ denotes the* topological *n-simplex*

$$\Delta^{n}=\left\{\left(p_{0},\ldots ,p_{n}\right)\in R^{n+1}\;\right|\;0\leq p_{i}\leq 1,\sum_{i}p_{i}=1$$

*The spaces $\Delta^{n},n\geq 0$ can be viewed as* cosimplicial *topological spaces with the following degeneracy and face maps:*

$$\delta_{i}\left(t_{0},\ldots ,t_{n}\right)=\left(t_{0},\ldots ,t_{i-1},0,t_{i},\ldots ,t_{n}\right)\;for\;0\leq i\leq n$$

$$\sigma_{j}\left(t_{0},\ldots ,t_{n}\right)=\left(t_{0},\ldots ,t_{j}+t_{j+1},\ldots ,t_{n}\right)\;for\;0\leq i\leq n$$

*Note that $\delta_{i}:R^{n}\to R^{n+1}$, whereas $\sigma_{j}:R^{n}\to R^{n-1}$.*

*The equivalence relation ∼ above that defines the quotient space is given as*

$$\left(d_{i}\left(x\right),\left(t_{0},\ldots ,t_{n}\right)\right)∼\left(x,\delta_{i}\left(t_{0},\ldots ,t_{n}\right)\right)$$

$$\left(s_{j}\left(x\right),\left(t_{0},\ldots ,t_{n}\right)\right)∼\left(x,\sigma_{j}\left(t_{0},\ldots ,t_{n}\right)\right)$$

### 7.3. Topological Embeddings as Coends

We will now bring in the perspective that topological embeddings of simplicial objects in cPROP categories can be interpreted as a coend [16] as well. Consider the functor

$$F:\Delta^{o}\times \Delta \to Top$$

where

$$F\left(\left[n\right],\left[m\right]\right)=X_{n}\times \Delta^{m}$$

where *F* acts *contravariantly* as a functor from Δ to **Sets** mapping $\left[n\right]\mapsto X_{n}$, and *covariantly* mapping $\left[m\right]\mapsto \Delta^{m}$ acts as a functor from Δ to the category Top of topological spaces.

The coend defines a topological embedding of a simplicial object *X* in a cPROP category, where $X_{n}$ represents composable morphisms of length *n*. Given this simplicial object, we can now construct a topological realization of it as a coend object

$$\int^{n}\left(X_{n}\right)\cdot \Delta n$$

where $X:\Delta^{op}\to C$ is the simplicial object defined by the contravariant functor from the simplicial category Δ into the category of simplicial objects in cPROP categories, and Δ:|Δ|→Top is a functor from the topological *n*-simplex realization of the simplicial category Δ into topological spaces Top. As MacLane [16] explains it picturesquely, the “coend formula describes the geometric realization in one gulp”. The formula says essentially to take the disjoint union of affine *n*-simplices, one for each $n\in X_{n}$, and glue them together using the face and degeneracy operations defined as arrows of the simplicial category Δ.

## 8. Homotopy in cPROP Categories

We define homotopy in cPROP categories somewhat abstractly in this section, but we will illustrate these definitions more concretely for Bayesian networks defined as functors between cPROP categories, which are analogous to the CDU functors [9] defined later in Section 9.

To motivate the need for considering homotopy in categorical models of causal inference, and in particular for cPROP categories, note that causal models can only be determined up to some equivalence class from data, and while many causal discovery algorithms assume that arbitrary interventions can be carried out, for example, on separating sets [6] and other types of subsets [7], to discover the unique structure, such interventions are generally impossible to do in practical applications. The concept of essential graph [52] and chain graph [53] are attempts to formulate the notion of a “quotient space” of graphs, but similar issues arise more generally for non-graph-based models as well. Thus, it is useful to understand how to formulate the notion of equivalent classes of causal models in an arbitrary category. For example, given the conditional independence structure A⊥⊥B|C, there are at least three different symmetric monoidal categorical representations that all satisfy this conditional independence [9,10,23], and we need to define the quotient space over all such equivalent categories.

### 8.1. Homotopy in cPROP Categories

We will discuss homotopy in cPROP categories more generally now. This abstract notion of homotopy generalizes the notion of homotopy in topology, which defines why an object like a coffee cup is topologically homotopic to a doughnut (they have the same number of “holes”).

**Definition** **26.** *Let C and $C^{\prime }$ be a pair of objects in a cPROP category C. We say C is a retract of $C^{\prime }$ if there exists maps $i:C\to C^{\prime }$ and $r:C^{\prime }\to C$ such that $r\circ i={id}_{C}$.*

**Definition** **27.** *Let C be a cPROP category. We say that a morphism f:C→D is a retract of another morphism $f^{\prime }:C\to D$ if it is a retract of $f^{\prime }$ when viewed as an object of the functor category* **Hom** *([1],C). A collection of morphisms T of C is closed under retracts if for every pair of morphisms $f,f^{\prime }$ of C, if f is a retract of $f^{\prime }$, and $f^{\prime }$ is in T; then, f is also in T.*

**Definition** **28.** *Let X and Y be simplicial cPROP categories represented as simplicial sets, and suppose we are given a pair of morphisms $f_{0},f_{1}:X\to Y$. A homotopy from $f_{0}$ to $f_{1}$ is a morphism $h:\Delta^{1}\times X\to Y$ satisfying $f_{0}{=h|}_{0\times X}$ and $f_{1}=h_{1\times X}$.*

### 8.2. Classifying Spaces of cPROP Categories

I now introduce a formal way to define causal effects in our cPROP framework, which relies on the construction of a topological space associated with the nerve of a cPROP category. As shown in [28], the nerve of a category is a full and faithful embedding of a category as a simplicial object.

**Definition** **29.** *The classifying space BC of a cPROP category C is the topological space associated with the nerve of the category C.*

To understand the classifying space BC of a cPROP category C, let us go over some simple examples to gain some insight.

**Example** **7.** *Consider a discrete cPROP category $C_{X}$ as a subcategory over* **FinSet** *defined as discrete finite sets X with no non-trivial morphisms, where the classifying space $BC_{X}$ is just the discrete topology over X (where the open sets are all possible subsets of X).*

**Example** **8.** *Consider a cPROP category C defined as a partially ordered set [n], with its usual order-preserving morphisms; then, the nerve of [n] is isomorphic to the representable functor Δ(−,[n]), as shown by the Yoneda Lemma, and in that case, the classifying space is just the topological space associated with $\Delta_{n}$ (the topological n-simplex). For the pancreatic cancer domain described in Section 2, if we view causal models as posets, then their classifying space is given by the topological n-simplex.*

### 8.3. Homotopy Colimits of cPROP Categories

**Definition** **30.** *The homotopy colimit of a cPROP category model is defined as a nerve of the category of elements associated with the set-valued functor δ:C→* **Set** *mapping the cPROP category C to a dataset, namely, $B\left(\int \delta \right)$.*

In general, we may want to evaluate the homotopy colimit of a cPROP category not only with respect to the data used in a causal experiment but also with respect to some underlying topological space or some measurable space. We can extend the above definition straightforwardly to these cases using an appropriate functor T: **Set** → **Top** or, alternatively M: **Set**→ **Meas**. These augmented constructions can then be defined with respect to a more general notion called the *homotopy colimit* [29] of a causal model.

**Definition** **31.** *The topological homotopy colimit ${hocolim}_{T\circ \delta }$ of a cPROP category C, along with its associated category of elements associated with a set-valued functor δ:C→* **Set** *and a topological functor T:* **Set** *→* **Top** *, is isomorphic to topological space associated with the nerve of the category of elements that is ${hocolim}_{T\circ \delta }≃B\left(\int \delta \right)$.*

**Example** **9.** *The classifying space ${BC}_{CDU}$ associated with CDU symmetric monoidal category encoding of a causal Bayesian DAG [9] is defined using the monoidal category (***C** , ⊗, *I), where each object A has a copy map $C_{A}:A\to A⊗A$, discarding map $D_{A}:A\to I$, and a uniform state map $U_{A}:I\to A$, which is defined as the topological realization of its nerve. As before, the nerve B(C) of the CDU (or Markov) category is defined as the set of sequences of composable morphisms of length n.*

$$\left\{C_{o}\overset{f_{1}}{\to }C_{1}\overset{f_{2}}{\to }\ldots \overset{f_{n}}{\to }C_{n}\;|\;C_{i}\;is\;an\;object\;in\;C,f_{i}\;is\;a\;morphism\;in\;C\right\}$$

*Note that the CDU category was associated with a CDU functor F: ${\mathrm{Syn}}_{G}\to$* **Stoch** *to the category of stochastic matrices. We can now define the homotopy colimit ${hocolim}_{F}$ of the CDU causal model associated with the CDU category C, along with its associated category of elements associated with a set-valued functor δ:C→* **Set** *, and a topological functor F:* **Set** *→* **Stoch** *is isomorphic to the topological space associated with the nerve of the category of elements over the composed functor, that is, ${hocolim}_{F\circ \delta }$.*

### 8.4. Defining Causal Effect in cPROP Categories Using Homotopy

Finally, we turn to defining causal effect using the notion of classifying space and homotopy colimits, as defined above. Space does not permit a complete discussion of this topic, but the basic idea is that once a causal model is defined as a topological space, there are a large number of ways of comparing two topological spaces from analyzing their chain complexes, or we can use a topological data analysis method such as UMAP [54].

**Definition** **32.** *Let the classifying space under “treatment” be defined as the topological space $BC_{1}|$ associated with the nerve of a cPROP category $C_{1}$ under some intervention, which may result in a topological deformation of the model (e.g., deletion of an edge). Similarly, the classifying space under “no treatment” be defined as the $BC_{0}$ under a no-treatment setting, with no intervention. A causally non-isomorphic effect exists between cPROP categories $C_{1}$ and $C_{0}$ or $C_{1}\;/\;≅C_{0}$ if and only if there is no invertible morphism $f:{BC}_{1}\to {BC}_{0}$ between the “treatment” and “no-treatment” topological spaces, namely, f must be both* left invertible *and* right invertible.

There is an equivalent notion of causal effect using the homotopy colimit definition proposed above, which defines the nerve functor using the category of elements. This version is particularly useful in the context of evaluating a causal model over a dataset.

**Definition** **33.** *Let the homotopy colimit ${hocolim}_{1}=B\int \delta_{1}$ be the topological space associated with a cPROP category $C_{1}$ under the “treatment’ condition defined with respect to an associated category of elements defined by a set-valued functor $\delta_{1}:C\to$* **Set** *over a dataset of “treated” variables, and let “no-treatment” ${hocolim}_{0}=B\int \delta_{0}$ be the topological space of a causal model associated with a cPROP category $C_{0}$ defined over an associated category of elements defined by a set-valued functor $\delta_{0}:C\to$* **Set** *over a dataset of “placebo” variables. A causally non-isomorphic effect exists between cPROP categories $C_{1}$ and $C_{0}$ or $C_{1}\;/\;≅C_{0}$ if and only if there is no invertible morphism $f:B\int \delta_{1}\to B\delta_{0}$ between the “treatment” and “no-treatment” homotopy colimit topological spaces, namely, f must be both left invertible and right invertible.*

## 9. Classifying Spaces of Bayesian Networks

In this section, we will drill down from the abstractions above to prove a set of more concrete results regarding the classifying spaces of cPROP functors that correspond to Bayesian networks [40] and that can be seen as analogous to CDU functors in affine CD categories [9]. In this section, we will restrict our attention to the cPROP category $c\underline{P}$ defined by the coalgebraic PROP $\underline{P}$ defined by the PROP maps δ:1→2 and ϵ:1→0, as discussed earlier in Section 4. We will also build on the results of the previous sections to state a categorical generalization of the Meek–Chickering (MC) theorem for cPROP categories [3,12]. This theorem, originally stated as a conjecture in Meek’s dissertation [12], was formally proved by Chickering [3]. The MC theorem states that, given any two causal DAG models G and H, H is an *independence map* of G that is any conditional independence implied by the structure of H that is also implied by the structure of G. Furthermore, there exists a finite sequence of edge additions and *covered* edge reversals such that after each edge change, G remains a DAG, H remains an independence map of G, and, finally, G=H after the sequence is completed.

**Figure 12:**
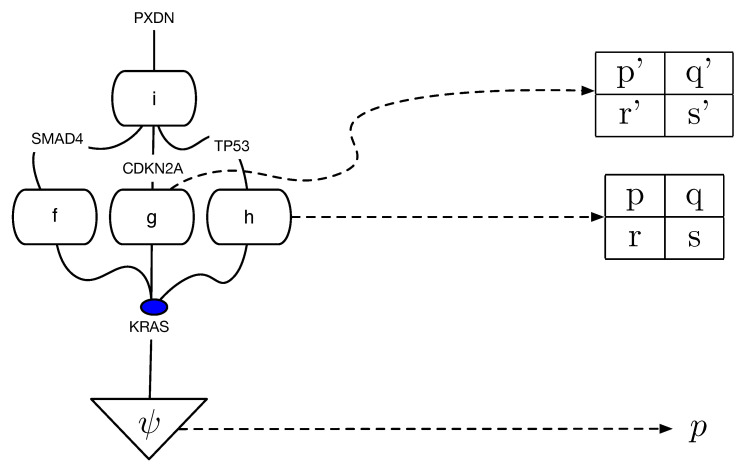
Example showing a cPROP functor modeling a causal model from the pancreatic cancer domain. The domain category is a string diagram represented as a Markov category [17]. The codomain category is **FinStoch**, the category of finite stochastic processes. The dashed arrows show how each morphism in the domain category is mapped to a corresponding conditional probability table, in the usual manner for Bayesian network models (each (i,j) entry defines the conditional probability of whether a gene has mutated or not, depending on the mutation status of the gene that precedes it in the partial ordering). Not all object or morphism mappings are shown: to fully specific a functor, each object and morphism in the domain category must be mapped into a suitable object and morphism in the codomain category.

To begin with, we build on the characterization of a causal DAGs G, or Bayesian networks [2,40], as functors from the cPROP (or equivalently CDU) category ${\mathrm{Syn}}_{G}$ to **FinStoch** (see [9] for more details). It is assumed that the reader is familiar with the terminology of DAG models in this section, and the reader is referred to [3] for additional details that have been omitted in the interests of space. A brief overview of the Markov category **FinStoch** is given (which was called **Stoch** in [9]), whose objects are finite sets and morphisms f:A→B. States are stochastic matrices from a trivial input I:={∗}, which are essentially column vectors representing marginal distributions. The counit is a stochastic matrix with a row vector consisting only of 1’s. The composition of morphisms is defined by matrix multiplication. The monoidal product ⊗ in **FinStoch** is the Cartesian product on the objects and Kronecker product of matrices ${\left(f⊗g\right)}_{\left(i,j\right)}^{\left(k,l\right)}:=f_{i}^{k}g_{j}^{l}$. The Kronecker product corresponds to taking product distributions. **FinStoch** realizes the “swap” operation defined by the string diagram in Definition 11 as σ:A⊗B→B⊗A given by $\sigma_{ij}^{kl}:=\delta_{i}^{l}\delta_{j}^{k}$, making it into a symmetric monoidal category. Figure 12 gives an example of such a functorial representation of Bayesian networks from the pancreatic cancer domain described in Section 2. The probability values shown in the figure are estimated frequencies from actual data in [38].

**Theorem** **11** (Proposition 3.1, [9])**.** *There is a 1–1 correspondence between Bayesian networks based on a DAG G and cPROP functors of the type F: ${\mathrm{Syn}}_{G}\to$* **FinStoch**.

This theorem is essentially the same as that in [9], since functors between the CDU categories ${\mathrm{Syn}}_{G}$ and **FinStoch** are special types of functors between cPROP categories. We can model the category of all Bayesian networks as a functor category ${\mathrm{FinStoch}}^{{Syn}_{G}}$ on cPROP categories. In this section, we will explore the homotopic structure of this functor category, whose objects are Bayesian networks represented as functors and whose arrows are natural transformations.

Let us now build on the homotopic structures defined earlier in Section 8 in terms of viewing each cPROP category C in terms of its classifying space BC. The following theorem is straightforward to prove.

**Theorem** **12.** *Each Bayesian network encoded as a cPROP functor F: ${\mathrm{Syn}}_{G}\to$* **FinStoch** *induces a continuous and cellular map of CW complexes (i.e., compactly generated spaces with a weak Hausdorff topology [55]).*

$$\mathrm{BF}:B{\mathrm{Syn}}_{G}\to B\mathrm{FinStoch}$$

**Proof.**  Recall that B is a functor from the category Cat to the category Top of topological spaces defined as the classifying space of a category, which is constructed by forming the simplicial set using the nerve of the category (where each *n*-simplex represents composable morphisms in a category of length *n*) and using its topological realization as defined by Milnor [30].    □

We can define an equivalence structure on cPROP functors representing DAG models, generalizing the classical definitions in Pearl [2] and using Theorem 11 above.

**Theorem** **13.** *Two cPROP functors $F_{1}:$ ${\mathrm{Syn}}_{G_{1}}\to$* **FinStoch** *and $F_{2}:$ ${\mathrm{Syn}}_{G_{2}}\to$* **FinStoch** *are* **equivalent** *and denoted as $F_{1}\approx F_{2}$, where we use the same symbol ≈ used in [3] for DAG equivalence if they are constructed from DAG models $G_{1}$ and $G_{2}$, respectively, that have the same skeletons and the same V-structures.*

**Proof.**  Two DAGs are known to be equivalent, meaning they are *distributionally equivalent* and *independence equivalent* if their *skeletons*, namely, the underlying undirected graph ignoring edge orientations, are isomorphic and have the same *V-structures*, meaning an ordered triple of nodes (X,Y,Z) where G contains the edges X→Y and Z→Y, and *X* and *Z* are not adjacent in G. Given that Theorem 11 gives us a 1–1 correspondence between the DAG models and cPROP functors, the theorem follows straightforwardly.    □

### 9.1. Natural Transformations Between Causal Models

I now introduce another significant concept from category theory—*natural transformations*—and use it to define the relationships between two causal models, such as Bayesian networks. In a range of situations in causal inference, from representing the effect of an intervention [2,9] to searching the space of DAG models in the GES algorithm [3], it is necessary to relate two causal models with each other. In the previous section, it has been shown how each causal model, such as a Bayesian network, can be viewed as a functor from a “syntactic” category to a “semantic” category. Here, the section will introduce how natural transformations between two functors capture the relationships between two Bayesian networks or other causal models.

**Definition** **34.** *Given any two functors F:C→D and G:C→D between the same pair of categories, a natural transformation from F to G is defined through a collection of mappings: one for each object c of C, thereby defining a morphism in D for each object in C. They are defined as follows:*

- *An arrow $\alpha_{c}:Fc\to Gc$ in D for each object c∈C, which together define the components of the natural transformation.*
- *For each morphism $f:c\to c^{\prime }$, the following commutative diagram holds true:*


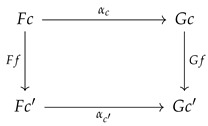

*A natural isomorphism is a natural transformation α:F⇒G in which every component $\alpha_{c}$ is an isomorphism.*

**Figure 13:**
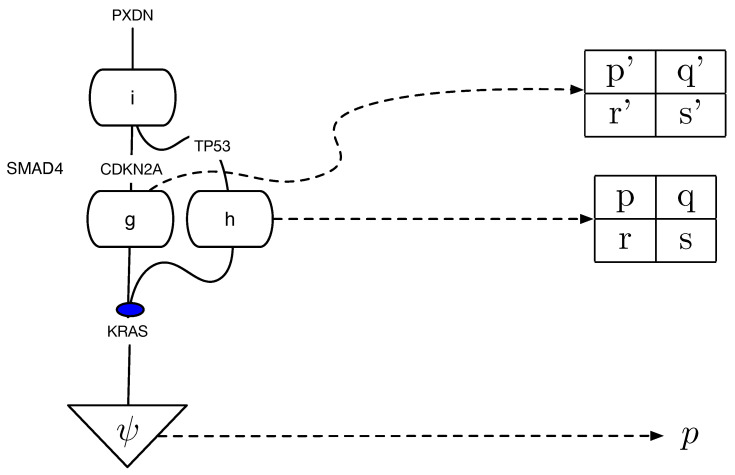
The functor *G* that defines a causal model resulting from modifying the original functor *F* in Figure 12 by an intervention that deletes the influence of the gene **SMAD4** on the mutation of gene **PXDN**.

To concretize this abstract notion of a natural transformation, let us define as the functor *F* the causal model shown in Figure 12, and let the functor *G* be defined by the causal model in Figure 13, which deletes the morphism from KRAS to SMAD4. In terms of the natural transformation, note that each path in the above commutative diagram defines a morphism in the codomain “semantic” category. For the specific deleted morphism *f*, note that Ff will continue to denote the original mapping from KRAS to SMAD4, but Gf will now be mapped into the empty mapping, as there is now no causal pathway in the intervened model.

To generalize from the example just given, if we want to define the set of all possible natural transformations between two causal models represented as functors, we can use an elegant framework introduced by Yoneda called (co)end calculus [16]. Specifically, we can characterize the interaction between two Bayesian networks represented as cPROP functors through Yoneda’s (co)end calculus, where for simplicity we use the same cPROP category ${\mathrm{Syn}}_{G}$ to denote that these DAGs have the same skeleton and V-structures.

**Theorem** **14.** *Given two cPROP functors $F_{1}:$ ${\mathrm{Syn}}_{G}\to$* **FinStoch** *and $F_{2}:$ ${\mathrm{Syn}}_{G}\to$* **FinStoch** *representing two DAG models, the set of natural transformations between them can be defined as an end* 

$${\mathrm{FinStoch}}^{{Syn}_{G}}\left(F_{1},F_{2}\right)=\int_{c}\mathrm{FinStoch}\left(F_{1}\left(c\right),F_{2}\left(c\right)\right)$$

**Proof.**  The proof of this result follows readily from the standard result that the set of natural transformations between two functors is an end (see page 223 in [16]).    □

We can this use this result to construct a homotopic structure on the topological space of all continuous and cellular maps of CW complexes defined in Theorem 12 above.

**Theorem** **15.** *The topological space of all continuous and cellular maps of CW complexes, where each map is defined as*

$$\mathrm{BF}:B{\mathrm{Syn}}_{G}\to B\mathrm{FinStoch}$$

*is decomposed into equivalence classes by the equivalence relation ≈ defined in Theorem 13.*

**Proof.**  The equivalence relation ≈ on cPROP functors is reflexive, symmetric, and transitive, because as Theorem 11 showed, there is a 1–1 correspondence between causal DAG models and cPROP functors. Each equivalence class of DAG models maps precisely into an equivalence class of cPROP functors.   □

**Theorem** **16.** *We can now bring to bear some properties of the classifying space developed by Segal [28] to construct a homotopy on cPROP categories and functors:*

- *For any two cPROP functors $F_{1}:$ ${\mathrm{Syn}}_{G}\to$* **FinStoch** *and $F_{2}:$ ${\mathrm{Syn}}_{G}\to$* **FinStoch** *, a natural transformation $\tau :F_{1}\Rightarrow F_{2}$ induces a homotopy between $BF_{1}$ and $BF_{2}$.*
- *If $F:{\mathrm{Syn}}_{G}\to {\mathrm{FinStoch}}_{G}$ and $G:{\mathrm{FinStoch}}_{G}\to {\mathrm{Syn}}_{G}$ is an adjoint pair of functors, then $B{\mathrm{FinStoch}}_{G}$ is homotopy equivalent to $B{\mathrm{Syn}}_{G}$ (here, ${\mathrm{FinStoch}}_{G}$ is a subcategory of FinStoch that is defined by the mapping of each object and morphism in ${\mathrm{Syn}}_{G}$).*

**Proof.** 

- We can think of the natural transformation τ as a functor $T_{G}$ from ${\mathrm{Syn}}_{G}\times \left[1\right]$ to ${\mathrm{FinStoch}}_{G}$. We define the action of $T_{G}$ on objects as $T_{G}\left(C,0\right)=F_{1}\left(C\right)$ and $T_{G}\left(C,1\right)=F_{2}\left(C\right)$. On morphisms $f\in {\mathrm{Syn}}_{G}\left(C_{1},C_{2}\right)$, we can set $T_{G}\left(f,1_{0}\right)=F_{1}\left(f\right)$ and $T_{G(}f,1_{1})=F_{2}\left(f\right)$. For the only non-trivial morphism 0<1 in [1], we define $T_{G}\left(1_{C},0<1\right)=\tau_{C}$. The composite structure
  $$B{\mathrm{Syn}}_{G}\times \left[0,1\right]\equiv B\left({\mathrm{Syn}}_{G}\times \left[1\right]\right)\overset{B(T_{G})}{\to }B{\mathrm{Stoch}}_{G}$$
  yields the desired homotopy.
- Given any adjoint pair of functors $F:{\mathrm{Syn}}_{G}\to {\mathrm{FinStoch}}_{G}$ and $G:{\mathrm{FinStoch}}_{G}\to {\mathrm{Syn}}_{G}$, we can define the induced natural transformations η:Id⇒GF and ϵ:FG⇒Id. From the just established results on the natural transformation τ, the desired homotopy follows.

    □

### 9.2. Generalizing the Meek–Chickering Theorem to cPROP Categories

Since cPROP functors are in 1–1 correspondence with DAG models from Theorem 11, we can associate with any covered edge in a DAG model G an equivalent covered morphism in the Markov category ${\mathrm{Syn}}_{G}$ associated with the DAG model G.

**Theorem** **17.** *Let G be any DAG model, with associated cPROP functor $F_{G}$, let G’ be the result of reversing the edge X→Y, and let $F_{G^{\prime }}:{\mathrm{Syn}}_{G^{\prime }}\to {\mathrm{Stoch}}_{G^{\prime }}$ be the corresponding modified cPROP functor. Then, there is an induced natural transformation corresponding to reversing an edge and $F_{G}\approx F_{G^{\prime }}$ using the definition of cPROP functor equivalence in Theorem 13 if and only if the edge X→Y is a covered edge in G.*

**Proof.**  The proof of this theorem follows readily from Lemma 2 in [3], showing that G’ is a DAG model that is equivalent to G if and only if the edge that is reversed, namely, X→Y is covered in G.    □

**Theorem** **18** ([3])**.** *Let $F_{G}$ and $F_{G^{\prime }}$ be a pair of equivalent cPROP functors corresponding to two equivalent DAG models G and $G^{\prime }$, for which there δ edges in G that have the opposite orientation in $G^{\prime }$. Then, there exists a sequence of δ corresponding natural transformations transforming the functor $F_{G}$ into the functor $F_{G^{\prime }}$, where natural transformation can be implemented by constructing the cPROP functor for each intervening DAG model that is based on reversing a single additional edge, satisfying the following properties:*

- *Each natural transformation in $F_{G}$ must correspond to a covered edge in G.*
- *After each natural transformation, the functors $F_{G}\approx F_{G^{\prime }}$.*
- *After all natural transformations are composed, the two functors $F_{G}\approx F_{G^{\prime }}$.*

**Proof.**  Once again, the proof follows readily from the equivalent Theorem 3 in [3] exploiting the isomorphism between causal DAG models and cPROP functors from Theorem 11.    □

To state the homotopic generalization of the Meek–Chickering theorem for functors between cPROP categories, we need to define the partial ordering on cPROP functors.

**Definition** **35.** *Define the partial ordering ${\mathrm{Syn}}_{G}\leq {\mathrm{Syn}}_{H}$ to indicate that the corresponding causal DAG H is an independence map of G. Here, ≤ implies that if G≤H, then by necessity H contains more edges than G.*

Once again, it follows from the 1–1 correspondence between Bayesian networks and cPROP functors that the corresponding cPROP category ${\mathrm{Syn}}_{H}$ must contain more morphisms than ${\mathrm{Syn}}_{G}$. We can now state the generalized Meek–Chickering theorem for functors between cPROP categories.

**Theorem** **19.** *Let ${\mathrm{Syn}}_{G}$ and ${\mathrm{Syn}}_{H}$ be cPROP categories corresponding to any pair of DAGs G and H such that G≤H. Let r be the number of edges in H that have the opposite orientation in G, and let m be the number of edges in H that do not exist in either orientation in G. These edges translate correspondingly to the differences in morphisms in ${\mathrm{Syn}}_{G}$ and ${\mathrm{Syn}}_{H}$. Then, there exists a sequence of at most r+2m natural transformations that map the cPROP functor $F_{G}$ into the cPROP functor $F_{H}$ satisfying the following properties:*

- *Each edge reversal and corresponding natural transformation corresponds to a covered edge.*
- *After each natural transformation corresponding to an edge reversal and edge addition, ${\mathrm{Syn}}_{G}\leq {\mathrm{Syn}}_{H}$.*
- *After all r+2m natural transformations are composed, $Syn_{G}\approx Syn_{H}$ is a natural isomorphism.*

**Proof.**  The proof generalizes in a straightforward way from Theorem 4 in [3], since we are exploiting the 1–1 correspondences between causal DAG models and cPROP functors. The proof of this theorem in [3] is constructive, since it involves an algorithm, and it would take more space than we have to sketch out the entire process of categorifying it. But, each step in the Algorithm APPLY-EDGE-ORIENTATION in [3] can be equivalently implemented for cPROP categories using the correspondences between causal DAGs and cPROP functors.    □

### 9.3. Homotopy Groups of Meek–Chickering Causal Equivalences

We can now define the equivalence classes under the Meek–Chickering formulation in a more abstract manner using abstract homotopy. First, we define the notion of an equivalence class of objects in any category C simply as that defined by the connectedness relation defined by the morphisms. Two objects *C* and $C^{\prime }$ are in the same equivalence class E in a category C if the following structure holds true:

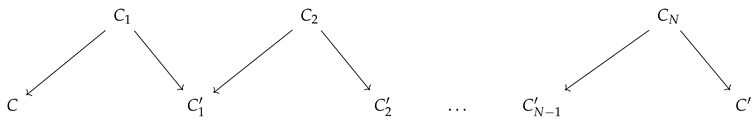

**Definition** **36.** *Define the set of path components of a category C as the set of equivalence classes of the morphism relation on the objects by $\pi_{0}C$.*

**Theorem** **20** ([29])**.** *The set of path components of the topological space BC, namely, $\pi_{0}\mathrm{BC}$, is in bijection with the set of path components of C.*

This relationship between the original category C and its topological realization BC now gives us a homotopic characterization of the GES algorithm described in Section 3. More formally, the GES proceeds by moving from one equivalence class of causal models to the next by addition or removal of (non-covered) edges. These steps can be characterized in terms of the natural transformations between equivalence classes of the cPROP (or CDU [9]) functors that define the causal DAGs. We treat the equivalence class of DAGs within each connected component as a locally connected topological space. Thus, the set $\pi_{0}C$ is exactly the number of equivalence classes, which is again the same as the number of connected components in $\pi_{0}\mathrm{BC}$, defining the 0*^th^* homotopy group in the topological realization of the category C.

**Theorem** **21.** *The GES procedure can be formally characterized topologically as moving from one equivalence class of connected topological spaces in BC to another, where an equivalence class of connected objects in BC is defined by the connectedness relation of natural transformations that correspond to reversals of covered edges within an equivalence class.*

**Proof.**  The proof of this theorem follows directly from Theorems 2 and 20 and its homotopic version stated as Theorem 19. □

To summarize the results of this section, it has been described how to construct the classifying spaces corresponding to Bayesian network causal models, which lets us construct a homotopic equivalence across causal models represented as functors on cPROP categories. Categorical generalizations of the definitions in [3] have been introduced, and the categorical generalization of the Meek–Chickering theorem for Markov categories has been stated.

## 10. Classifying Spaces of cPROP Categories

We now return to a more abstract discussion of the classifying spaces BC of a general cPROP category C. As a cPROP category, C is a symmetric monoidal category, and there is a wealth of known results that can be brought to bear on its homotopic structure; a full discussion of this topic is beyond the scope of this introductory paper. I want to give the reader a taste of the many approaches that can be brought to bear on the structure of equivalence classes in causal models. First, I want to discuss the connection between the multiplicative structure of symmetric monoidal categories, like Markov categories, and the commutative *H*-space structure on BC, which is its classifying space.

**Definition** **37** ([29])**.** *An H-space is a topological space X with a chosen base point $x_{0}$ (which in cPROP categories can be associated with the topological realization of the terminal object) and a continuous map μ:X×X→X such that the maps $\mu (x_{0},.)$ and $\mu (.,x_{0})$ are homotopic to the identity map on X with respect to homotopies that preserve the basepoint $x_{0}$. An H-space is associative if μ is associative up to homotopy, and it is commutative if μ is commutative up to homotopy.*

**Definition** **38** ([29])**.** *An H-space X is group-like if there is a continuous map χ:X→X such that $\mu \circ (1_{id}\times \chi )\circ \Delta$ is homotopic to the identity, where* Δ *is the the diagonal map on X.*

It is worth mentioning that the comonoidal structure of a cPROP category C induces a diagonal map Δ:BC→BC×BC on its topological realization through the nerve through the (uniform) copy map ${\mathrm{copy}}_{X}:X\to X⊗X$.

**Theorem** **22.** *Let C be a cPROP category. Then, its classifying space BC is an associative and commutative H-space.*

**Proof.**  The proof of this theorem is based on a simple diagram chase, building on the standard result for (small) symmetric monoidal categories.

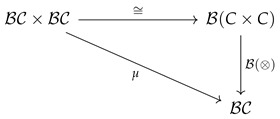

The complete proof can be seen in [29] (Theorem 13.1.4). □

**Theorem** **23.** *The classifying space BC of a small cPROP category C is contractible.*

**Proof.**  This result follows from the fact that the tensor unit element *I* in a cPROP category is a terminal element, implying that there is a unique morphism ${\mathrm{del}}_{X}:X\to I$, as discussed previously. Using the result from Theorem 16, we exploit the fact that there is an adjoint pair of functors from C to [0] and the topological realization B[0] is a point. □

We can define the higher homotopy groups of a cPROP category C as follows.

**Definition** **39.** *Given a cPROP category C and an arbitrary object X in C, for n≥0, the $n^{th}$ homotopy group of C with respect to the basepoint X is defined as*

$$\pi_{n}\left(X,C\right)=\pi_{n}\left(\mathrm{BC},\left[X\right]\right)$$

*where [X] is the 0-simplex associated with the basepoint X.*

## 11. A Higher Algebraic K-Theory of cPROP Categories

Finally, we build on the above results to give a formal higher algebraic K-theory of causal inference in terms of the causal equivalence classes. To make the construction concrete, we begin with the notion of Grothendieck group completion defined by the Grayson–Quillen construction. Here, the intuition is to begin with an Abelian monoid and make it into a universal group by adding “inverse” elements to each monoid element. The result is a characterization of the K-theory structure.

### 11.1. Grayson-Quillen Group Completion

The basic idea for a “K-theory” arose from the notion of a Grothendieck “K” group that came out from his proof of the Riemann–Roch theorem, which involved the analysis of isomorphism classes of objects of an Abelian category with a tensor product (direct sum). This specific construction was later generalized in the following way. Consider a commutative monoid *M* for which the most general Abelian group *K* needs to be constructed by building “inverse” elements to all the elements of *M*. Such a “group completion” always exists and is characterized by a universal property

i:M→K,

where *i* is a monoid homomorphism mapping *M* to the group *K* that satisfies the universal property that for any other monoid homomorphism

f:M→A,

there is a unique group homomorphism

g:K→A

such that f=g∘i. To show the relevance of this construction to causal inference, we briefly outline the Grayson–Quillen K-theory for symmetric monoidal categories Grayson [56] that we can use to associate with any cPROP category C an augmented category $C^{-1}C$ using a particular group completion method.

**Definition** **40.** *Let (C,⊗,e,τ) be a small symmetric monoidal category. Denote by $C^{-1}C$ the category whose objects are pairs of objects of C. Morphisms in $C^{-1}C$ from $\left(C_{1},D_{1}\right)$ to $\left(C_{2},D_{2}\right)$ are equivalence classes of pairs of morphisms*

$$\left(f:C_{1}⊗E\to C_{2},g:D_{1}⊗E\to D_{2}\right)$$

*where E is an object of C. Such pairs of morphisms are equivalent to*

$$\left(f^{\prime }:C_{1}^{\prime }⊗E^{\prime }\to C_{2},g^{\prime }:D_{1}⊗E^{\prime }\to D_{2}\right)$$

*if there is an isomorphism $h\in C(E,E^{\prime })$ such that the following diagram commutes:*

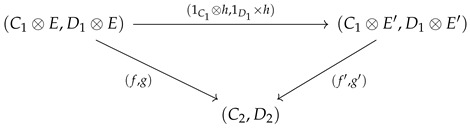


*The category $C^{-1}C$ is called the Grayson–Quillen construction of C.*

Note that this construction depends on the choice of object *E*. If *E* is selected to be the unit element *I* of a cPROP (or Markov category), then every pair of morphisms in C gives rise to a morphism in $C^{-1}C$.

**Theorem** **24** ([29], Lemma 13.3.2)**.** *The category $C^{-1}C$ is symmetric monoidal as well, and there is a lax monoidal functor $j:C\to C^{-1}C$, and $\pi_{0}\left({BC}^{-1}C\right)$ is an Abelian group.*

**Definition** **41.** *The K-Theory space associated with a cPROP category C is the classifying space $\mathrm{KC}={BC}^{-1}C$, where the $n^{th}$ K-group of C is its $n^{th}$ homotopy group $\pi_{n}{BC}^{-1}C$. Specifically, the fundamental group $\pi_{0}\left({BC}^{-1}C\right)$ is the Grothendieck group completion of the Abelian monoid induced by the path components $\pi_{0}\left(C\right)$.*

Let us now connect this procedure with the 0*^th^* homotopy group of the Meek–Chickering equivalence class.

**Theorem** **25.** *Let the cPROP category C correspond to causal DAG models, that is, each object is a functor representing a Bayesian network, and the arrows are natural transformations representing the corresponding covered edge reversals within a class. These connected components represent Meek–Chickering equivalence classes. Then,*

$$K_{0}\left(C\right)=\pi_{0}\left(\mathrm{KC}\right)=≃G_{0}\left(\pi_{0}\left(C\right)\right)$$

**Proof.**  This theorem states that the 0*^th^* order homotopy group corresponding to the Meek–Chickering equivalence classes is isomorphic to the Grothendieck group completion of the Abelian monoid $\pi_{0}\left(C\right)$. The proof follows readily from the more general result that holds in any symmetric monoidal category (see Lema 13.3.4 in [29]). □

### 11.2. cPROP Groupoids and Their Classifying Spaces

A groupoid is a category whose every morphism is invertible. We can characterize the Meek–Chickering causal equivalences in terms of the classifying spaces of their induced groupoids.

**Definition** **42.** *Define the Moore–Chickering groupoid as the cPROP category $G_{MC}$ whose objects are defined as the equivalence classes defined by the Moore–Chickering theorem and whose invertible morphisms correspond to covered edge reversals that map from an object back to itself.*

We can now give a simple characterization of the Moore–Chickering equivalence classes in terms of the classifying space of their induced groupoids.

**Theorem** **26.** *The classifying space of the Moore–Chickering groupoid category ${BG}_{MC}$ is defined as*

$${BG}_{MC}=\underset{i}{⨆}{BG}_{MC}^{i}$$

*where the disjoint sum index i ranges over equivalence classes.*

## 12. Causal Inference in Open Games and Network Economics

This section will briefly describe more advanced applications of the causal framework to open games [36], using a categorical compositional model that extends traditional Nash non-cooperative game theory [57], as well as network economics [37]. Both open games and network economics can be modeled using string diagrams in symmetric monoidal (Abelian) categories, which makes these applications amenable to analysis using the theoretical framework. A full discussion is beyond the scope of this paper, but I will provide sufficient detail here to explain how these applications can be modeled. Game theory has been used for many decades to model interactions among economic players. Billions of dollars of products, such as auctions of radio frequency bands by governments worldwide, have been sold using the principles of game theory. However, traditional game theory is non-compositional: a simple Prisoners Dilemma game between two players cannot be combined with another instance of such a game to create a larger game. In fact, it is not obvious that such a compositional approach is even possible.

A remarkable outcome of applying category theory to game theory is the compositional framework of *open games* [36]. The basic idea underlying compositional game theory is to model a game as a symmetric monoidal category. This formulation requires one crucial idea called *lenses* that has been previously used in categorical models of databases and deep learning [58]. Essentially, in addition to modeling each player’s move, as in traditional game theory, compositional game theory also adds the novel idea of a *coplay*, which is an extra mechanism that is used to transmit information backwards from one game theory module to its antecedent game module.

Network economics [37] has been extensively used to model games involving the trade of some electronic, monetary, or physical goods across a network. The underlying mathematics used to solve network economics games uses variational inequalities (VIs) [59]. We will briefly examine how to model a network economy as an open game and then describe how our cPROP causal framework applies to model causal interventions in network economics. An earlier paper of mine [60] explored causal inference in variational inequalities, without using the symmetric monoidal categorical framework described in this paper. Here, I introduce briefly the idea of a categorical framework for network economies.

### 12.1. Open Games

Ghani et al. [36] introduced the definition of an open game:

**Definition** **43.** *Let X, S, Y, and R denote sets of a game G. An open game G:(X,S)→(Y,R) is defined as a 4-tuple $G:(\Sigma_{G},P_{G},C_{G},B_{G})$ such that the following are determined:*

- *$\Sigma_{G}$ is the set of strategy profiles of G.*
- *$P_{G}:\Sigma_{G}\times X\to Y$ is the play function of game G.*
- *$C_{G}:\Sigma_{G}\times X\times R\to S$ is called the coplay function of G.*
- *$B_{G}:X\left(Y\to R\right)\to Rel\left(\Sigma_{G}\right)$ is defined as the best response function of G, where $Rel(\Sigma_{G})$ is a meet-semilattice of all endo-relations $R\subseteq \Sigma_{G}\times \Sigma_{G}$.*

What is crucial about this definition is that it is composable, meaning that we can form the sequential and parallel composition of open games to form larger open games, which is a crucial advantage that is not possible in traditional Nash games. I relegate the details to [36] but give the basic gist of the idea here before proceeding to outline how it can be used to define network economies as a symmetric monoidal category.

**Definition** **44.** *Given a pair of open games G:(X,S)→(Y,R) and H:(Y,R)→(Z,Q), the sequential composition H∘G:(X,S)→(Z,Q) is defined as follows:*

- *The set of strategy profiles is the Cartesian product $\Sigma_{H\circ G}=\Sigma_{G}\times \Sigma_{H}$.*
- *The play function for the composite game H∘H is simply the composition of the individual play functions:*
$$P_{H\circ G}\left(\left(\sigma ,\tau \right),x\right)=P_{H}\left(\tau ,P_{G}\left(\sigma ,x\right)\right)$$
- *The coplay function for the composite game is defined as*
$$C_{H\circ G}\left(\left(\sigma ,\tau \right),x,q\right)=C_{G}\left(\sigma ,x,C_{H}\left(\tau ,P_{G}\left(\sigma ,x\right),q\right)\right)$$
- *Finally, the best response relation of the composite game is defined as $\left(\left(\sigma ,\tau \right),\left(\sigma^{\prime },\tau^{\prime }\right)\right)\in B_{H\circ G}\left(x,k\right)$ that holds iff $\left(\sigma ,\sigma^{\prime }\right)\in B_{G}\left(x,k^{\prime }\right)$ and $\left(tau,tau^{\prime }\right)\in B_{H}\left(P_{G}\left(\sigma ,x\right),k\right)$, where $k^{\prime }:Y\to R$ is defined as*
$$k^{\prime }\left(y\right)-C_{H}\left(\tau ,y,k\left(P_{H}\left(\tau ,y\right)\right)\right)$$

**Figure 14:**
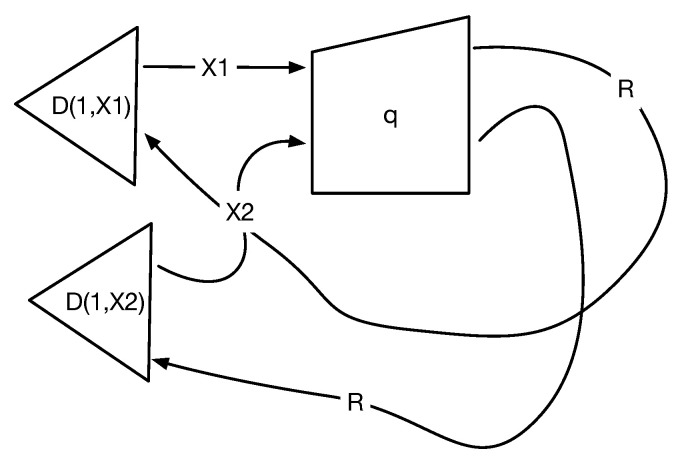
A string diagram of a symmetric monoidal category of a simultaneous move game [36].

One can similarly define the parallel composition of open games, wherein I will refer the reader to [36] for the requisite details. Figure 14 illustrates a string diagram representation of a symmetric monoidal category of a simultaneous move open game. The causal framework can now be applied to analyze equivalence classes in such open games, since these are now essentially string diagrams of the type we have illustrated previously in the paper. For example, a causal intervention in an open game might involve changing the payoff structure, which will involve a change in the game’s Nash equilibrium. I will discuss a more complex example below involving a multistage economic game on a network. We can apply our analysis of the classifying spaces of open games under causal interventions, which is a problem for future work.

### 12.2. Causal Inference in Variational Inequalities

I now give a brief introduction to variational inequalities (VIs) [59], which represent a mathematical framework used to solve problems in network economics [37]. VIs were originally developed in physics in the mid-1960s for modeling equilibrium problems in continuum mechanics, which generalizes= (convex) optimization, non-cooperative games, fixed point equation solving, and complementarity problems. VIs have been applied to a wide range of problems in network economics [37]. A VI is usually defined as a set of deterministic multidimensional vector field mappings $F_{i}:K_{i}\to R^{n}$, where $K_{i}$ is a convex set defined over a set of exogenous non-manipulable variables *U* and a set of endogenous modifiable variables *V*. The ensemble of vector field mappings jointly defines a global vector field mapping $F:K\to R^{n}$. Solving a variational inequality means finding an element x∈K where the set of inequalities holds:

(6) $$\langle F\left(x^{*}\right),\left(x-x^{*}\right)\rangle \geq 0,\;\;\forall x\in K$$

where 〈.,.〉 denotes the inner product in $R^{n}$. Variational inequalities were originally developed for infinite-dimensional Hilbert spaces. We consider only *n*-dimensional Euclidean spaces in this paper. Either setting can be modeled in terms of an enriched symmetric monoidal Abelian category [16]. To see the connection with optimization, note that if F(x)=∇f(x), the gradient of a (convex) multidimensional function, then the above condition is precisely the requirement for $x^{*}$ to be the (global) minimum.

**Figure 15:**
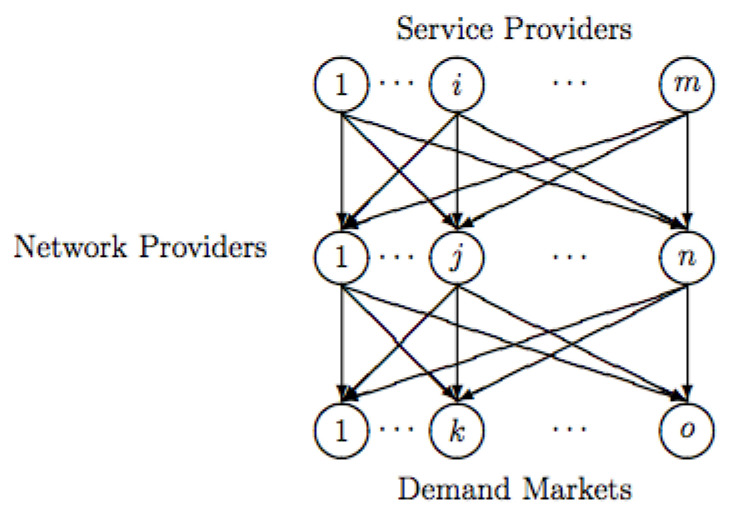
A generic network economic model based on [37]. Demand markets consisting of individual users or groups of users who choose combinations of service providers and transport providers, both of whom compete with each other for demand markets.

Broadly speaking, we will interpret causal intervention as inducing a submodel mapping $F_{w}$, which usually may be due to the manipulation of some endogenous variable *w*. However, our framework is agnostic on the particular manipulation mechanisms and extends to other possibilities, such as modifying a pricing function or a change in demand. As a simple example to build intuition, consider a network economy consisting of *m* producers of personal protective equipment (PPE) and *n* demand markets (see Figure 15). For trade to occur between the producer *i* and the demand market *j*, the following equilibrium condition must be satisfied:

(7) $$\begin{array}{ccc} \pi_{i}\left(Q\right)+c_{ij}\left(Q\right) & = & \rho_{j}\;\mathrm{if}\;\;Q_{ij}^{*}>0 \end{array}$$

(8) $$\begin{array}{ccc} \pi_{i}\left(Q\right)+c_{ij}\left(Q\right) & \geq & \rho_{j}\;\mathrm{if}\;\;Q_{ij}^{*}=0 \end{array}$$

This condition asserts that trade, measured by $Q_{ij}$ between the producer *i* and demand market *j*, will occur precisely when the supply price $\pi_{i}$ plus the transportation cost $c_{ij}$ is equal to the demand price $\rho_{j}$; otherwise, $Q_{ij}^{*}=0$. Note that the supply price and transportation costs are a function of trade volume *Q* over the whole network. We can write this equilibrium condition in the form of a variational inequality as follows:

$$\left(\pi_{i}\left(Q\right)+c_{ij}\left(Q\right)-\rho_{j}\right)\left(Q_{ij}-Q_{ij}^{*}\right)\geq 0$$

Note that if $Q_{ij}^{*}>0$, then according to Equation (7), the first term above must equal 0. Alternatively, if $Q_{ij}^{*}=0$, then the reason is that the combined cost of manufacturing $\pi_{i}$ and transportation $c_{ij}$ exceeds the demand price $\rho_{j}$; hence, the inequality is again satisfied. We can view each equilibrium condition above as a submodel $F_{ij}\left(Q\right)$, and there will be one such submodel for each producer and consumer. The number of submodels is equal to the number of trade paths, which collectively define the overall vector field mapping *F*. We can now model causal interventions in this system, such as raising or lowering prices, as inducing a modified model $F_{w}$, and analyze the effect of these causal interventions on the equilibrium trade between producers and suppliers. If we view the imposition of tariffs as a causal intervention, then the previous equilibrium solution of the network economy reflected in the amount of goods sold from producers to consumers and shipped via transporters will change, as recent events have shown. The causal intervention now requires recomputing a new solution to the multiplayer game represented by the network economy.

### 12.3. Example of a Network Economy

We will now describe a concrete example of a network economics problem. Network economics games can be considerably more complex than the single-layer games discussed in the previous section (see Figure 15) [37]. This network economics model comprises three tiers of agents: producer agents who want to sell their goods, transport agents who ship merchandise from producers, and demand market agents interested in purchasing the products or services. The model applies both to electronic goods, such as video streaming, token streaming for vendors of large language models (LLMs), and physical goods, such as smartphones or cars. This approach has also been used to model tariffs [61].

The model assumes *m* service providers, *n* network providers, and *o* demand markets. Each firm’s utility function is defined in terms of the non-negative service quantity (Q), quality (q), and price (π) delivered from service provider *i* by network provider *j* to consumer *k*. The production costs, demand functions, delivery costs, and delivery opportunity costs are designated by *f*, ρ, *c*, and oc, respectively. Service provider *i* attempts to maximize its utility function $U_{i}^{1}\left(Q,q^{*},\pi^{*}\right)$ by adjusting $Q_{ijk}$ (Equation (9a)). Likewise, network provider *j* attempts to maximize its utility function $U_{j}^{2}\left(Q^{*},q,\pi \right)$ by adjusting $q_{ijk}$ and $\pi_{ijk}$ (Equation (9b)).

(9a) $$\begin{array}{cc} U_{i}^{1}\left(Q,q^{*},\pi^{*}\right) & =\sum_{j=1}^{n}\sum_{k=1}^{o}{\hat{\rho }}_{ijk}\left(Q,q^{*}\right)Q_{ijk}-{\hat{f}}_{i}\left(Q\right)\\ \; & -\sum_{j=1}^{n}\sum_{k=1}^{o}\pi_{ijk}^{*}Q_{ijk},\;Q_{ijk}\geq 0 \end{array}$$

(9b) $$\begin{array}{cc} U_{j}^{2}\left(Q^{*},q,\pi \right)= & \sum_{i=1}^{m}\sum_{k=1}^{o}\pi_{ijk}Q_{ijk}^{*}\\ - & \sum_{i=1}^{m}\sum_{k=1}^{o}\left(c_{ijk}\left(Q^{*},q\right)+oc_{ijk}\left(\pi_{ijk}\right)\right),\\ \; & q_{ijk},\pi_{ijk}\geq 0 \end{array}$$

We assume the governing equilibrium is Cournot–Bertrand–Nash and the utility functions are all concave and fully differentiable. This establishes the equivalence between the equilibrium state we are searching for and the variational inequality to be solved, where the *F* mapping is a vector consisting of the negative gradients of the utility functions for each firm. Since *F* is essentially a concatenation of gradients arising from multiple independent, conflicting objective functions, it does not correspond to the gradient of any single objective function.

(10a) $$\begin{array}{cc} \; & \langle F\left(X^{*}\right),X-X^{*}\rangle \geq 0,\forall X\in K,\\ \mathrm{where}\;\; & X=\left(Q,q,\pi \right)\in R^{3mno+}\\ \mathrm{and}\;\; & F\left(X\right)=(F_{ijk}^{1}\left(X\right),F_{ijk}^{2}\left(X\right),F_{ijk}^{3}\left(X\right)) \end{array}$$

(10b) $$\begin{array}{cc} F_{ijk}^{1}\left(X\right) & =\frac{\partial f_{i}\left(Q\right)}{\partial Q_{ijk}}+\pi_{ijk}-\rho_{ijk}-\sum_{h=1}^{n}\sum_{l=1}^{o}\frac{\partial \rho_{ihl}\left(Q,q\right)}{\partial Q_{ijk}}\times Q_{ihl} \end{array}$$

(10c) $$\begin{array}{cc} F_{ijk}^{2}\left(X\right) & =\sum_{h=1}^{m}\sum_{l=1}^{o}\frac{\partial c_{hjl}\left(Q,q\right)}{\partial q_{ijk}}\;\;\;\;\;\;\;\;\;\;\; \end{array}$$

(10d) $$\begin{array}{cc} F_{ijk}^{3}\left(X\right) & =-Q_{ijk}+\frac{\partial oc_{ijk}\left(\pi_{ijk}\right)}{\partial \pi_{ijk}}\;\;\;\;\;\;\;\;\;\;\; \end{array}$$

The variational inequality in Equation (10a) represents the result of combining the utility functions of each firm into standard form. $F_{ijk}^{1}$ is derived by taking the negative gradient of $U_{i}^{1}$ with respect to $Q_{ijk}$. $F_{ijk}^{2}$ is derived by taking the negative gradient of $U_{j}^{2}$ with respect to $q_{ijk}$. And $F_{ijk}^{3}$ is derived by taking the negative gradient of $U_{j}^{2}$ with respect to $\pi_{ijk}$.

Note that a causal intervention, such as an imposition of tariffs, will require that Equation (10a) be resolved to reflect the new costs incurred in the production or shipment of goods, and that, in turn, will be reflected in the change in demand.

### 12.4. Causal Inference in Network Economics

We can apply our cPROP framework to network economics by converting the network economy in Figure 15 into an open game symmetric monoidal category with an associated string diagram, where causal inferences will now be reflected as “string diagram surgery” [9]. The new solution of the resulting VI will quantitatively measure the impact of the causal intervention. We can apply the framework of open games described above to the network economics framework as illustrated in Figure 15, where each node in the network (producer, transporter, or consumer) is viewed as a player in a network game. Each player is then modeled as shown in Figure 14, where the sequential and parallel composition of players in the network game is modeled in terms of the sequential and parallel composition in a symmetric monoidal category. To apply our higher-algebraic K-theory causal framework, we must note that there may be many equivalent string diagrams that are all causally equivalent to each other, and our theoretical results will help determine a more compact representation of this exponentially large space. A full analysis of this complex real-world domain is beyond the scope of this paper and is a topic for a future paper.

## 13. Summary and Future Work

In this paper, I analyzed the homotopic structure of observationally equivalent causal models using a categorical structure called cPROPs, which is a functor category from a coalgebraic PROP *P* to a symmetric monoidal category C. Such functor categories define the right adjoint of the inclusion of Cartesian categories in the larger category of all symmetric monoidal categories. cPROPs are an algebraic theory in the sense of Lawvere [20]. cPROPs relate closely to previous categorical models, such as Markov categories [17] and affine CDU categories [9,25], which can be viewed as a special type of cPROP defined by the PROP maps δ:1→2 and ϵ:1→0 that satisfies a set of commutative diagrams. To obtain topological insight into observationally equivalent classes of causal models, I characterized the classifying spaces of cPROPs by constructing their simplicial realization through the nerve functor. As a concrete application to causal inference, I showed that causal DAG equivalence generalizes to induce a homotopic equivalence across observationally equivalent cPROP functors. I presented a homotopic generalization of the Meek–Chickering theorem on causal equivalence in DAG models, where I viewed covered edge reversals connecting causally equivalent DAGs in terms of natural transformations between homotopically equivalent cPROPs. These results are a small sampling of the wide range of tools available in abstract homotopy theory, which I briefly describe next.

### 13.1. Operads and Iterated Loop Spaces

There is a rich set of theoretical results that connect the K-theory classifying spaces of symmetric monoidal categories and cPROP and Markov categories, with the geometry of iterated loop spaces and operads [62]. In particular, it is known that all connective spectra are based on some symmetric monoidal category and that special types of monoidal categories called permutative categories that yield Barrat–Eccles operads as their classifying spaces, which are a type of $E_{\infty }$ operad. A full discussion of these connections is a topic for future papers.

### 13.2. Model Categories on cPROP Categories

A standard approach to performing abstract homotopy on a category is to define its associated model structure. One way to do that implicit in our work is through its simplicial object structure. The category of simplicial sets is well known to have a rich model structure, which has been extensively studied [63]. This construction requires partitioning the space of morphisms into *fibrations*, *cofibrations*, and *weak equivalences* to construct a model category structure for causal inference [64]. I plan to devote a separate paper on this topic.

### 13.3. cPROPs for Non-Graphical Causal Models

I have restricted our attention in this paper to defining cPROPs for causal directed acyclic graph (DAG) models, as these are the most popular representation studied in previous work on categorical causality [9,10,14,23]. But, the general framework of cPROPs can be easily extended to non-graphical representations, such as integer-valued multisets (or imsets) [8]. For the specific case of a DAG model G=(V,E), an imset in standard form [8] is defined as

$$u_{G}=\delta_{V}-\delta_{\emptyset }+\sum_{i\in V}\left(\delta_{{\mathrm{Pa}}_{i}}-\delta_{i\cup {\mathrm{Pa}}_{i}}\right)$$

where each $\delta_{V}$ term is the characteristic function associated with a set of variables *V*. Finally, a separoid [65] is an algebraic framework for characterizing conditional independence as an abstract property, which is defined by a join semi-lattice equipped with a partial ordering ≤ and a ternary property ⊥⊥ over triples of elements such that X⊥⊥Y|Z defines the property that *X* is conditionally independent of *Y* given *Z*. One can define cPROPs for separoids as well.

## Figures and Tables

**Table 1:** A hallmark of cancerous tumors is their *genotype* of mutations of particular genes.

Tumor	Gene A	Gene B	Gene C	Gene D	⋯
Tumor 1	0	1	1	0	⋯
Tumor 2	1	0	1	1	⋯

**Table 2:** Core signaling pathways and processes genetically altered in most pancreatic cancers [33].

Regulatory Pathway	% Altered Genes	Tumors	Representative Altered Genes
Apoptosis	9	100%	CASP10, VCP, CAD, HIP1
DNA damage control	9	83%	ERCC4, ERCC6, EP300, TP53
G1/S phase transition	19	100%	CDKN2A, FBXW7, CHD1, APC2
Hedgehog signaling	19	100%	TBX5, SOX3, LRP2, GLI1, GLI3
Homophilic cell adhesion	30	79%	CDH1, CDH10, CDH2, CDH7, FAT
Integrin signaling	24	67%	ITGA4, ITGA9, ITGA11, LAMA1
c-Jun N-terminal kinase	9	96%	MAP4K3, TNF, ATF2, NFATC3
KRAS signaling	5	100%	KRAS, MAP2K4, RASGRP3
Regulation of invasion	46	92%	ADAM11, ADAM12, ADAM19
Small GTPase-dependent	33	79%	AGHGEF7, ARHGEF9, CDC42BPA
TGF-β signaling	37	100%	TGFBR2, BMPR2, SMAD4, SMAD3
Wnt/Notch signaling	29	100%	MYC, PPP2R3A, WNT9A

## Data Availability

Data used in this article are available from cited references as noted in the paper.
